# The dynamics of large silicic systems from satellite remote sensing observations: the intriguing case of Domuyo volcano, Argentina

**DOI:** 10.1038/s41598-020-67982-8

**Published:** 2020-07-15

**Authors:** Paul Lundgren, Társilo Girona, Mary Grace Bato, Vincent J. Realmuto, Sergey Samsonov, Carlos Cardona, Luis Franco, Eric Gurrola, Michael Aivazis

**Affiliations:** 1 Jet Propulsion Laboratory, California Institute of Technology, Pasadena, CA USA; 2 Canada Centre for Mapping and Earth Observation, Natural Resources Canada, Ottawa, Canada; 3 Observatorio Vulcanológico de Los Andes del Sur (OVDAS), Servicio Nacional de Geología y Minería, Temuco, Chile; 4 Parasim Inc., Pasadena, CA USA

**Keywords:** Natural hazards, Geophysics, Volcanology

## Abstract

Silicic magmatic systems are the most dangerous volcanoes on Earth, capable of large and catastrophic eruptions, yet their low eruptive frequency makes it challenging to interpret their short-term unrest. Here we present a decade-plus analysis that integrates, for the first time, time series of satellite interferometric synthetic aperture radar (InSAR) surface deformation and satellite thermal infrared edifice-scale surface warming at a large silicic system: Domuyo volcano, in Argentina. We find that deformation and warming are highly correlated, and depending on the sign and lag between the time series, either shallow sealing or magma influx could drive Domuyo’s ongoing inflation (~ 0.15 m/year; from an InSAR-derived tabular source, ~ 11 × 8 × 1 km; ~ 6.5 km depth; ~ 0.037 km^3^/year volume-change rate) and warming (0.3–0.4 °C/year). This study shows the potential that combined satellite surface deformation and edifice-scale surface warming time series have on assessing the physical mechanisms of silicic volcanic systems and for constraining deterministic models.

## Introduction

Volcanic unrest has traditionally (pre-twenty-first century) been discovered either through direct observations by local populations or through the analysis of *in-situ* data by observatory personnel. Thus, studies are usually focused on frequently active volcanoes or those in better instrumented locations (e.g., Kilauea or Etna). The detection of volcanic unrest has been greatly expanded thanks to the advent of satellite-based remote-sensing methods, casting light on the variability of volcanic unrest signals from both geodesy^1,2^ and thermal infrared spectroscopy^3–7^.

Over the past 20 years, satellite synthetic aperture radar interferometry (InSAR) has discovered previously unrecognized volcanoes undergoing unrest^1,8,9^. In many of these volcanoes, deformation has been detected without other unrest signals, either due to deformation preceding other signs of activity or because there was no significant observational infrastructure in place to identify other signals. Deformation unrest can be variable, with continuous inflation and deflation at Campi Flegrei, Italy^10^, and Yellowstone^11^ calderas, or with sudden onset of inflation at long-dormant systems such as Lazufre^8^, South Sister^9^, and Laguna del Maule^12^. Lacking other observations (e.g., thermal, gas, seismicity), the simplest interpretation of deformation unrest is that it represents magma migration at depth. InSAR spatial deformation patterns, as well as the time dependence of the deformation, allows the constraint of source model parameters that form the basis for time variable physical model interpretation^13–15^.

Satellite-based thermal infrared (TIR) remote sensing of volcanoes has a history of over half a century^5,16^. The detection of thermal unrest is achieved through two different approaches. The first is by detecting and tracking hotspots (e.g., using ASTER^6^, MODIS-based automatic platforms such as MODVOLC^3^ or MIROVA^4^; or SEVIRI-based automatic platforms such as HOTVOLC^17^), typically associated with fumaroles or the presence of a magma body at the surface^3,18^. This approach has permitted reporting of possible short-term (< 1 year) pre-eruptive thermal anomalies at a number of volcanoes, including ones in Kamchatka, Russia^19–21^; Asama, Japan^22^; Etna, Italy^23^; Hudson, Chile^24^; and Eyjafjallajökull*,* Iceland^25^. The second, recently developed, approach^7^ consists of computing the long-term (> 1 year), large-scale (10 s-to-100 s of km^2^), variations of the volcano’s median ground brightness temperature using the MODIS instruments aboard NASA’s Terra and Aqua satellites, which are thought to reflect the enhancement of subsurface hydrothermal activity. This technique has been shown to be efficient at detecting long-term pre-eruptive surface warming for different types of eruptions (including magmatic, phreatic, and hydrothermal), even for volcanoes where no other unrest signals have been reported.

**Figure 1:**
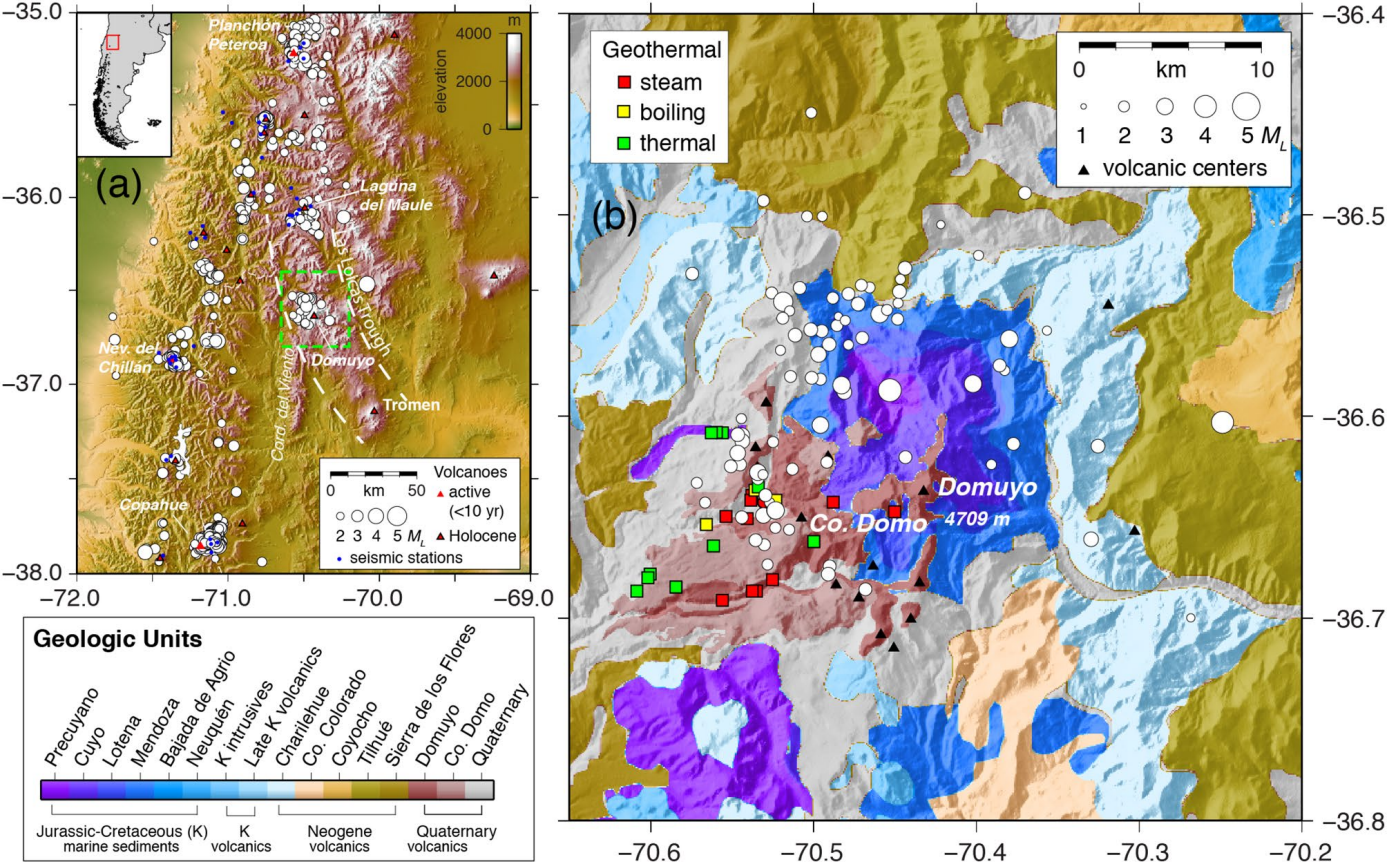
Domuyo volcanic complex. (**a**) Domuyo volcano lies in the southern Andes in Argentina, east of the main volcanic arc. Seismicity (white circles) greater than M_L_ 2 at Domuyo is comparable to other active volcanoes (Copahue, Nevados del Chillán, Planchon Peteroa, Laguna del Maule). Green dashed box around Domuyo volcano is the area covered in (**b**) and the interferograms shown in Fig. 2. (**b**) Seismicity (white circles) scaled by magnitude, geothermal springs, and volcanic centers plotted over a simplified geological map based on Galetto et al.^26^. Purple to darker blue represent older marine sediments, whereas light blue through earth colors are Miocene-Quaternary volcanic rocks (legend, lower left).

In this work, we present decade-plus observations of surface deformation and large-scale surface warming at a large silicic system: Domuyo volcano. Domuyo is an intriguing volcanic complex located in northern Patagonia, Argentina (Fig. 1). It is the largest such complex in the Las Loicas Trough, which also includes Laguna del Maule caldera to the NW and Tromen volcano to the SE^26^. Geologically, Domuyo is a young volcano composed of a complex of Tertiary rhyolitic to dacitic intrusions, overlain by Quaternary pyroclastic deposits and lava flows that intrude and overlie back arc shallow Mesozoic marine deposits^26^. Domuyo is recognized mostly for its geothermal potential, which is centered beneath its southwest flanks, extending approximately 15 km from the summit^27^. A hydrochemical study of the geothermal field^25^ found up to 1.1 GW thermal energy release, making it second only to Yellowstone in advective geothermal heat flux. They concluded that such a large heat flux is hard to explain in terms of the cooling of magmatic intrusions dated at 0.72 Ma or the most recent magmatic eruptive activity at 0.11 Ma from Cerro Domo^26^, thus suggesting recent reactivation of the system. Reactivation of Domuyo is also supported by several gas-driven explosions occurring in the active hydrothermal system over the past two decades^27,28^. A chemical and isotopic analysis^29^ found evidence for both shallow (400–600 m) and deeper (2–3 km) hydrothermal reservoirs, as well as evidence for an actively degassing magma source at unknown depth. They considered the high (~ 86%) mantle He contribution surprising for a system with no eruptive activity in 100 ka. A new analysis of gravity, magnetic, InSAR surface deformation, and geomorphology data at Domuyo found that recent (2015–2018) inflation is consistent with volatile rich magma intruded at 7 km depth^30^.

In the following, we show how the dynamic processes governing the current unrest of Domuyo, a silicic system without a magmatic eruption in ~ 0.1 Ma, can be deciphered combining time series of surface deformation and edifice-scale surface warming. Specifically, we compile InSAR surface deformation from 2008–2019 (derived from ALOS, RADARSAT-2, ALOS-2, and Sentinel-1 satellites) and surface warming from 2002–2019 (derived from the moderate resolution imaging spectroradiometers -MODIS- aboard NASA’s Aqua and Terra satellites). InSAR time series are used to constrain a continuum mechanical model for an ellipsoidal volume source, whose consistency is tested by comparing with the location, timing and moment release of recent seismicity. Surface warming time series is used to compare the current warming levels with those preceding the last two gas-driven eruptions in the active hydrothermal field (2007 and 2012), explore its correlation with deformation, and then infer feasible conceptual deformation-warming models. Finally, we discuss the status of Domuyo volcano in the context of these conceptual models, as well as the implications they have for processes of long-term silicic volcano reservoir evolution and for possible future eruptions.

## Results

We present the results from analysis of InSAR data and geodetic source modeling, TIR surface warming time series, and cross-correlation of the two time-series. The methodology and analysis details behind these results are given in the Methods section and Supplementary table and figures.

### Surface deformation and source modelling

**Figure 2:**
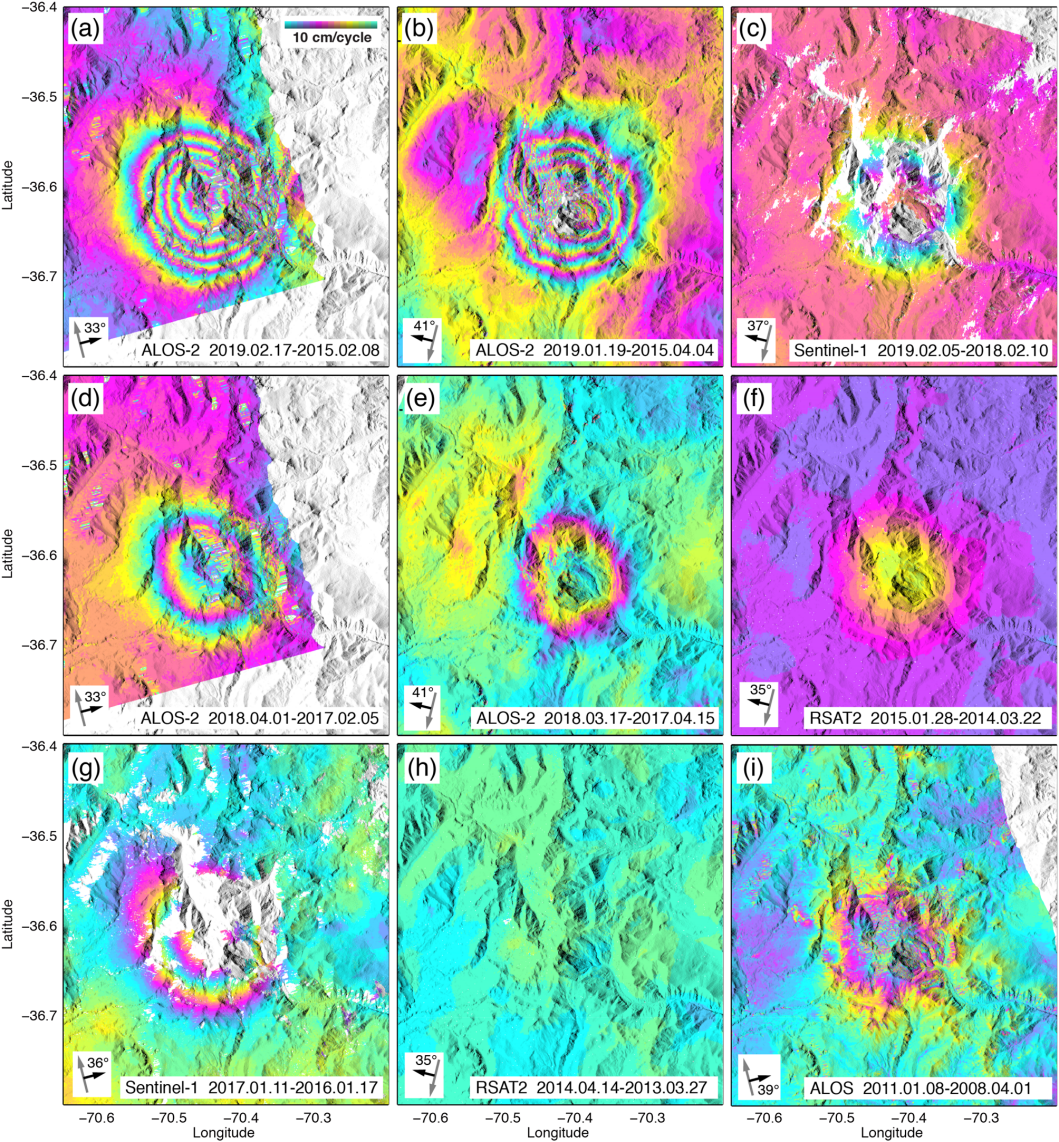
Unwrapped SAR interferograms. Image area corresponds to dashed box in Fig. 1a. In each image, the satellite heading (gray arrow) and look direction (black arrow) and ground incidence angle from zenith are shown in the lower left corner. Bottom label in each gives the satellite and master–slave dates. All interferograms are re-wrapped at 10 cm per color cycle. (**a**, **b**) ~ 4-year interferograms from ALOS-2 showing cumulative inflation patterns. (**c**, **d**, **e**, **g**) Example 1-year interferograms from ascending and descending track ALOS-2 and Sentinel-1 satellite data. (**f**) RADARSAT-2 10-month interferogram spanning the start of inflation. (**h**) RADARSAT-2 interferogram showing null deformation from early 2013 into 2014. (**i**) ALOS nearly 3-year interferogram showing deflation from 2008–2011.

**Figure 3:**
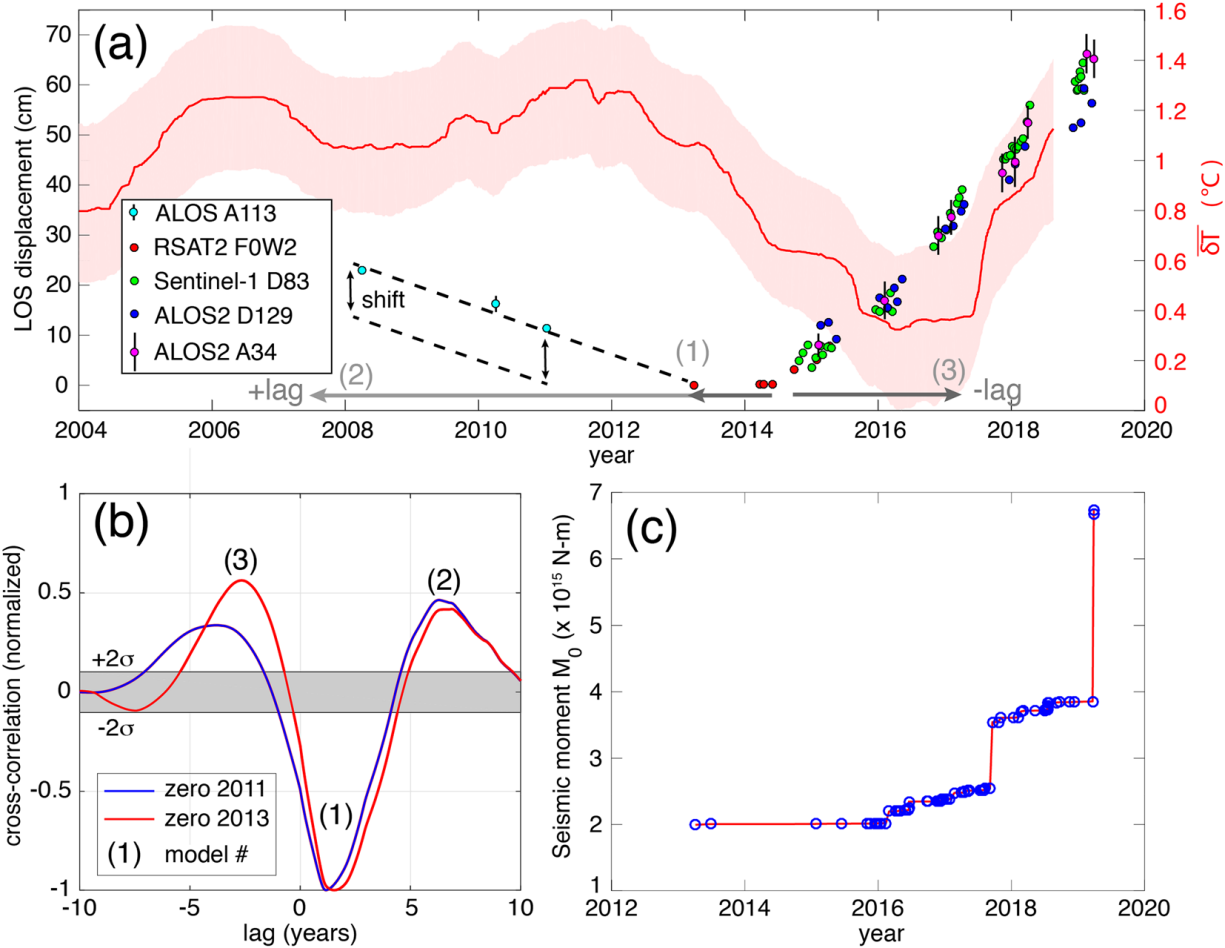
Integration of several data. (**a**) InSAR plus thermal time series. The individual InSAR time series and their 95% error bars (where visible) were manually adjusted relative to the RADARSAT-2 (RSAT2) time series, with the black dashed lines and vertical arrows showing the range of shifts possible for the ALOS time series that do not overlap in time with the RSAT2 observations. The thermal time series is given by the red line and pink 95% confidence bounds. The light and dark gray arrows indicate the possible time lags of the thermal relative to the InSAR time series, with the gray number in parenthesis corresponding to the model number and correlation peak shown in panel (**b**). (**b**) Normalized cross-correlation between the mean-removed surface deformation and mean-removed warming at Domuyo volcano (from early 2008 to mid-2018, when both time series are available). Cross-correlation measures the similarity between the surface deformation time series and shifted copies of the surface warming time series as a function of the lag. Normalization is performed with respect to the maximum similarity obtained (in absolute value). Gray band represents the two-standard deviation (95%) confidence interval, defined here by the maximum cross-correlation (in absolute value) obtained between 10,000 zero-mean Gaussian functions and the surface warming time series. Cross-correlation is calculated using Matlab. (**c**) Cumulative seismic scalar moment, *M*_*0*_, for earthquakes from the Volcano Observatory of the Southern Andes (OVDAS). Locations and density of seismic stations, mostly in Chile, prevent detection of events smaller than about *M*_*L*_ 2.

Radar interferograms from different radar satellite missions (ALOS, RSAT2, Sentinel-1, ALOS-2; see Methods for details) and different time intervals show a sequence of deflation and null deformation followed by the current on-going inflation (Fig. 2). Individual interferograms (Supplementary Figs. 1–6) from each satellite track are used to compute InSAR time series (Supplementary Fig. 7). In particular, RSAT2 data reveal that there was no resolvable deformation between 2013 and early 2014, and from mid-2014 to September 2014, there was an abrupt onset of inflation (within the temporal sampling of the data). We temporally connect the RSAT2 time series to the roughly linear inflation that has continued into 2019 (Fig. 3a) by manually adjusting the time series overlaps. The combined plot (Fig. 3a) of different satellites with differing look directions, incidence angles, and points selected for the time series plot, is suitable for showing the relative deformation behavior to first order. In the case of the ALOS time series, there is no temporal overlap with the RSAT2 time series’ zero deformation in early 2013. Therefore, the plot of the ALOS time series in Fig. 3a is imposed to intersect zero deformation in early 2013, coincident with the start of the RSAT2 time series, since it appears a shorter period of null time series change is in better agreement with the thermal time series null interval from late-2015 to mid-2017. In any case, this choice does not affect our interpretation and discussion (see Methods, where we compare the implications of whether the ALOS time series reaches zero deformation in early 2011 or early 2013).

We model the ongoing inflation of Domuyo using the cascading adaptive transitional metropolis in parallel (CATMIP) algorithm, implemented in the Caltech-developed AlTar software and generalized at JPL for kinematic and time variable fault and volcano modeling^31^. AlTar was applied employing the compound dislocation model^32^ (CDM), which represents a finite ellipsoidal volume change source composed of three orthogonal dislocations. The solution for the CDM source model constrained by the 2015–2019 ALOS-2 ascending and descending linear velocity data set (Supplementary Figs. 8–11) is an oblate (pancake-like) tabular source, gently tilted toward the west (see values in Supplementary Table 1), with depth centered at ~ 6.5 km below the surface. The volume influx rate (*ΔV/Δt*), or potency rate, where *ΔV* = *4u(ab* + *bc* + *ac*) and *Δt* is the inflation time, is ~ 0.0373 ± 0.0010 (2σ) km^3^/year.

Our geodetic analysis is complemented with the location map of seismic activity in the area around Domuyo (Fig. 1) and the time series of scalar moment (*M*_*0*_; Fig. 3c). Compared to the geodetic source (Supplementary Fig. 11), we see a pattern of seismicity falling in a semi-anulus around the ellipsoidal source (Supplementary Fig. 11b).

### Surface warming

The TIR time series analysis shows that the thermal output of Domuyo volcano (Fig. 3a) featured relatively small undulations from 2004 to 2012, with two maxima around thermal anomaly $\overline{\delta T} = 1.2 - 1.3^{ \circ } {\text{C}}$ that coincides with the onset of the last two gas-driven explosions at the active ‘El Humazo’ hydrothermal field^28^. Since the beginning of 2012, the thermal emissions declined at a roughly steady rate (~ $- 0.25^{ \circ } {\text{C}}/{\text{year}}$) through late 2016, with an abrupt increase of thermal emissions over the ~ 1.5 years from ~ mid-2017 through late 2018 ($0.3 - 0.4{ }^{ \circ } {\text{C}}/{\text{year}}$). Note that the thermal emissions beyond late 2018 cannot be retrieved due to the edge effects produced by the filtering technique applied to remove seasonality and noise^7^, although the most recent value of the anomaly retrieved is $\overline{\delta T} = 1.1 \pm 0.3^{ \circ } {\text{C}}$, close to the values observed prior to the 2007 and 2012 gas driven explosions.

### Surface deformation versus surface warming

Visual inspection of the evolution of surface deformation and surface warming suggests a possible link between both time series at Domuyo (Fig. 3b). This possible link is assessed through a cross-correlation analysis over the 10-year period (early 2008 to mid-2018) when both time series are available (see Methods section). Cross-correlation analysis shows a high degree of similarity between both time series at three specific time-shifts: first, surface warming is anti-correlated with surface deformation and precedes it by ~ 1.5 years. Second, the two time series are positively correlated; inflation/deflation precedes warming/cooling of the volcanic edifice by ~ 2.7 years. Third, the time series are positively correlated with warming/cooling of the volcanic edifice preceding inflation/deflation by ~ 6.9 years. Independently of the relative importance of the different correlation peaks, which may be affected by the short duration and non-stationarity nature of the time series, this similarity analysis between surface deformation and warming helps us constrain the possible subsurface processes governing the current unrest episode of Domuyo volcano (see Discussion).

## Discussion

### What do the combination of InSAR and TIR time series tell us about the system?

The integration of geodesy and thermal infrared spectroscopy using cross-correlation analysis allow us to explore possible mechanisms for the current unrest episode of Domuyo. These mechanisms are probably relevant to interpret surface deformation and warming at other systems as well, especially restless calderas^10,33^.

We find three primary correlation magnitudes with opposite lags and signs (Fig. 3b): (1) a positive lag with negative correlation amplitude, which implies that the InSAR time series follows the thermal time series by ~ 1.5 years with opposite sign; (2) a positive lag with positive correlation amplitude, which implies that the InSAR time series follows the thermal time series by ~ 6.9 years with same sign; and (3) a negative time lag with positive correlation amplitude, which implies that the thermal increase that began in mid-2017 follows the InSAR observed inflation starting in mid-2014 by ~ 2.7 years. These three scenarios give rise to three candidate models to explain the observed deformation and warming time series described below.

#### Thermal leads geodetic with negative correlation: time-varying permeability model

A positive lag (thermal leads InSAR) with negative correlation could imply a top-down process controlled by permeability changes of the shallow crust. In such a case, the period from 2008 to 2012 is explained by the permeable transport of magmatic gases from the reservoir towards the surface, which enhances shallow hydrothermal activity and warming of the surface^7^, and depressurizes the reservoir, thus yielding the warming and deflation of the volcanic edifice. A sudden decrease of the effective permeability (i.e., ‘sealing’) of the shallow subsurface, coinciding approximately with the 2012 austral winter hydrothermal explosion, would inhibit the transport of gas from the reservoir, thus resulting in reduction of hydrothermal activity, pressure increase in the reservoir, and corresponding cooling (from 2012 to 2016) and inflation (ongoing since 2014) of the ground surface. In this model, the ~ 1.5-year delay represents the time required for the shallow reservoir to over-pressurize significantly due to the accumulation of gas that cannot efficiently escape from the magma body. When the reservoir pressure increases sufficiently, pore pressure and permeability increase, porous gas flow and hydrothermal activity are enhanced again, and thus the surface gets warmer anew (ongoing since 2017). This implies that inflation should stop in the near future and the volcano should enter into a new deflation phase soon.

A time-varying permeability model would keep Domuyo active without the need for magma injections into the shallow reservoir. In fact, a possibility is that no magma injections have occurred at Domuyo since its last extrusive magmatic activity about 100 ka ago, which implies that the volume of fresh volatile-rich magma that was emplaced in the shallow crust is not yet depleted of exsolved volatiles. The volatile depletion time of a magma body can be estimated with $\alpha \rho_{m} V/Q$, where $\alpha$ is the mass fraction of volatiles that can exsolve from the magma at reservoir depths, $\rho_{m}$ is the magma density, $V$ is the volume of the magma body, and $Q$ is the average outgassing flux. Assuming realistic values for the different parameters ($\alpha \le 4\%$, $\rho_{m} = 2,400 {\text{kg}}/{\text{m}}^{3}$, $Q \ge 1 {\text{Kg}}/{\text{s}}$), we find volatile depletion times of $\sim 30 - 300$ ka for a magma body volume in the range $V = 10^{10} - 10^{11} {\text{m}}^{3}$. This would mean that the volume of volatile-rich magma emplaced beneath Domuyo after its last extrusive magmatic activity was, at least, about the same order of magnitude of the magma reservoir volume of Laguna del Maule^34^. Note, however, that there are some important caveats to consider: (i) It is unlikely that Domuyo has been emitting magmatic gases and heat at the current rates (it is the second most energetic volcano-hydrothermal system in the world after Yellowstone^27^) without more or less regular magma injections. This is so because passive outgassing during quiescence can lead to significant depressurizations in magma reservoirs, which destabilizes the hydraulic and mechanical equilibrium of the shallow crust, thus fostering magma ascent from the deep plumbing system^35–37^. (ii) It is unclear what mechanism can quickly decrease the effective permeability of the shallow subsurface around 2012. Processes such as mineral precipitation in the pore space are slow^38,39^, whereas the effect of the austral winter hydrothermal explosion on permeability is expected to be very local (due to the very low magnitude of the explosion). In addition, if they have any effect, explosions in the hydrothermal field are expected to open pathways for gas transport and thus increase, instead of decrease, the permeability. (iii) The current ~ 5-year inflation phase (ongoing since 2014) should mimic the ~ 4-year cooling phase spanning from 2012 to 2016. Inflation phases are not expected to last longer than their leading cooling phases, thus suggesting that the behavior of Domuyo during the period under study is not dominated by permeability variations.

#### Thermal leads geodetic with positive correlation: volatile release at depth

A positive lag (thermal leads InSAR) with positive correlation could imply a bottom-up process controlled by deep CO_2_ release from magma rising from the mantle^40,41^. Carbon dioxide exsolves at high pressures and percolates towards the surface in the early stages of magma migration and reactivation^39^. This supplies heat to the shallow hydrothermal system of Domuyo, which could enhance fluid circulation and potentially produce boiling in underground aquifers, with the subsequent condensation of steam and release of latent heat beneath the soil^42^; this could lead to warming of the surface before the magma reaches the shallow crust and the ground deforms elastically. In this model, the ~ 6.9-year delay represents the time required for magma to reach the shallow reservoir relative to the time required for CO_2_ to enhance hydrothermal activity and increase surface thermal emissions. This implies that the current inflation phase of Domuyo, ongoing since 2014, is indeed reflecting the warming period from 2008 to 2012; hence, the volcano should enter into a new deflation phase soon to reflect the cooling period from 2012 to 2016.

There are, however, some caveats to consider when applying this model to Domuyo: (i) The ~ 6.9-year positive lag with positive correlation reflects the similarity between the edges of the thermal and deformation time series, and thus it may just represent an artifact of the cross-correlation analysis. (ii) The current ~ 5-year inflation phase (ongoing since 2014) should mimic the ~ 4-year warming phase spanning from 2008 to 2012 (note that the longer thermal infrared time series allows us to infer that warming began around 2008 (Fig. 3a)). The current inflation phase would be expected to last no longer than leading warming phases, thus suggesting that the correlation between the deformation and thermal time series of Domuyo during the period under study is not dominated by volatile release at depth.

#### Geodetic leads thermal with positive correlation: porous flow-induced delay

A negative lag (InSAR leads thermal) with positive correlation could imply a reservoir-driven process controlled by the injection of magma into the shallow reservoir and the slow permeable transfer of gases from the reservoir to the surface. In particular, we propose that the ~ 2.7-year time lag observed corresponds to the time required for the magmatic gases to rise from the reservoir to the shallow subsurface, interact with the hydrothermal system, and warm the surface^7^.

A porous flow-induced delay model yields values of the effective permeability of the crust that are consistent with typical estimates (see Methods for theoretical basis). In addition, this model is in agreement with observations at restless calderas such as Campi Flegrei, where gas emission time series have been found to lag geodetic time series for large uplifts^43^. These large uplifts are interpreted as magmatic, whereas lower rates of inflation are considered largely gas-driven^10^. Similar to Campi Flegrei, if inflation at Domuyo stops without an eruption, it would be expected to give way to deflation as gas percolates out of the system.

Under this model, the 2008–2013 InSAR deflation prior to the current inflation at Domuyo would represent gas percolation for an interval of unknown duration. This geodetic deflation would correspond to the thermal cooling from 2012 to 2016, thus we would expect that geodetic data prior to 2008 would have lower amplitude inflation/deflation undulations similar to the decade of thermal time series before 2012. Considering our three candidate models arising from the cross-correlation analysis between deformation and warming time series, the third model, geodetic leading thermal due to porous flow-induced delay, appears to be the most physically viable.

### Towards a unifying model

**Figure 4:**
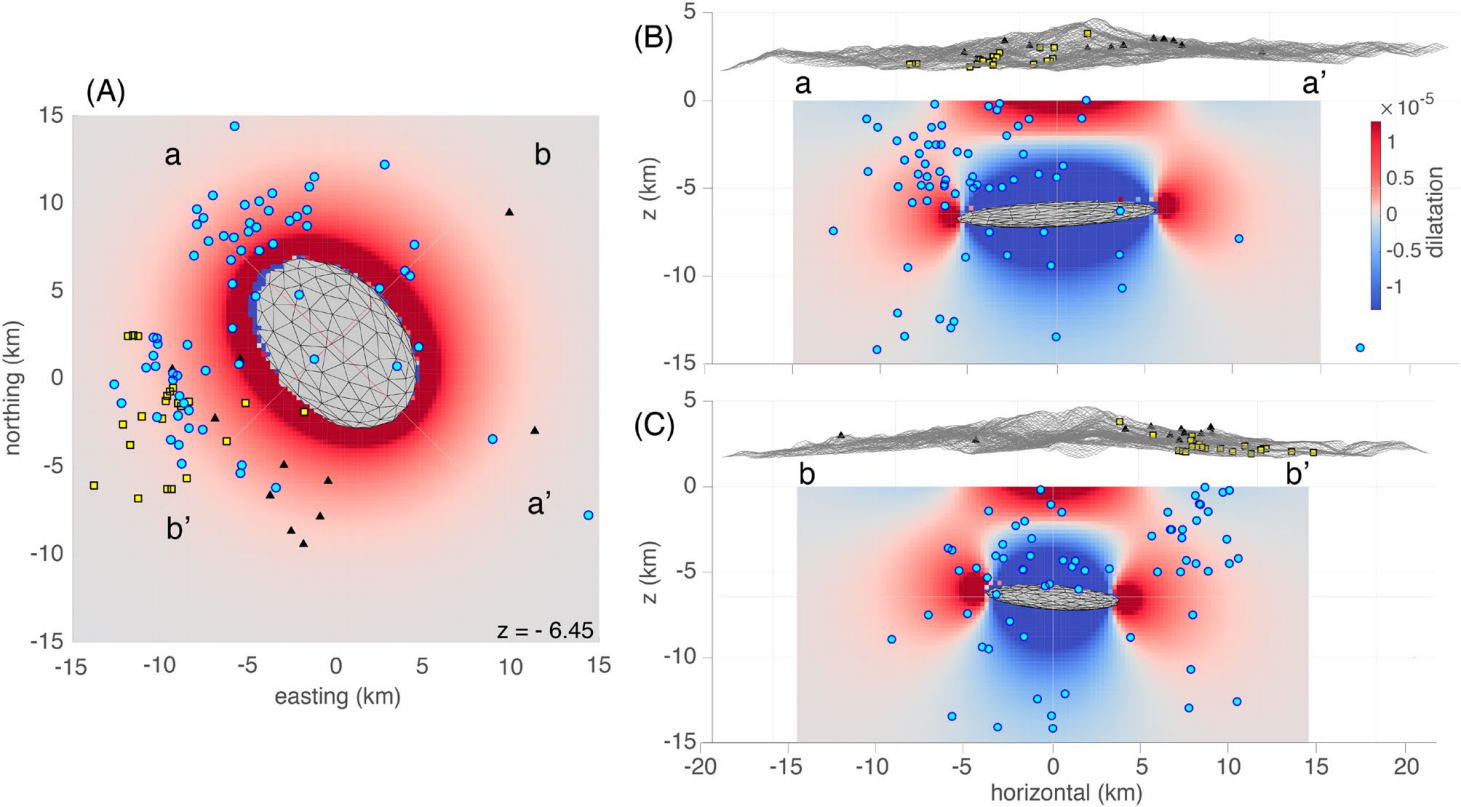
Dilatation (trace of the strain tensor) computed for and ellipsoidal cavity with *a, b, c* axes from the AlTar solution (Table S1) using the boundary element method (BEM) modeling software *cutanddisplace*^71^. Gas percolation is expected to be enhanced in areas of positive dilatation (warm colors) and inhibited in areas of negative dilatation (cool colors). (**A**) Map view for a horizontal slice at 6.45 km depth (same depth as ellipsoidal cavity center). Seismicity is as shown in Fig. 1b. Events deeper than 6.45 km depth are made visible. Here we see that seismicity generally falls in areas of positive dilatation. (**B**) Horizontal view from the SW, perpendicular the plane striking parallel the *a*-axis. (**C**) Horizontal view from the NW, perpendicular the plane striking parallel the *b*-axis. For (**B**) and (**C**), seismicity behind the vertical plane are made visible. Seismicity that appears to be directly above the source in the “blue” areas of negative dilatation are mostly away from the source such that they fall within areas of positive dilatation when viewed from the orthogonal direction, either (**C**) or (**B**).

The porous flow-induced delay model (model 3) provides a feasible explanation for the correlation between the InSAR and thermal time series. In addition, from the InSAR derived model, we can compute the dilatation rate (trace of the strain-rate tensor) assuming an overpressure rate of ~ 0.25–0.30 MPa/year. Although the magnitude of stressing rate is directly related to the source dimensions, the pattern of strain is not sensitive to the stress magnitude, and Lamé parameters of 30 GPa each (Fig. 4). Although such a model does not consider regional stresses, local structures, nor hydrothermal system effects on gas transport, it does provide a first-order agreement with the location of small earthquakes, occurring predominantly in a ring around the deformation source in areas of positive dilatation rate. In addition, this model suggests that gas percolation is a broad-scale phenomenon that is enhanced towards the edges of the ellipsoid (Fig. 4b), thus enhancing a broad-scale interaction with hydrothermal systems. This is consistent with an edifice-scale thermal warming signal.

The deflation-inflation behavior of the system over the past decade suggests a volcanic system with similarities to observations at restless calderas such as Campi Flegrei, Italy^10^, and Yellowstone, USA^11^. Such systems can have significant variations in the duration and intensity of deflation and inflation episodes, sometimes with eruptions, that are attributable to the steady growth of caldera resurgence^33^. Beyond conceptual models for surface manifestations of magma influx at depth, physics-based laboratory and numerical models suggest that magma emplacement history and the flux of magmatic episodes play roles in determining whether magma remains trapped in the upper crust or leads to dike propagation to the surface^44,45^. Scaled laboratory models^45^ suggest that the threshold for propagating dikes that lead to eruption is around 1 m^3^/s, or about 0.03 km^3^/year. This is approximately the influx rate we infer for Domuyo (< 0.037 km^3^/year), and also similar to that of nearby Laguna del Maule^15,46^ (~ 0.03–0.04 km^3^/year). It is intriguing that a volcanic system with no recent eruptive activity, and therefore with no expectations that our new observations of volcanic unrest spell an imminent eruption, is subjected to influx rates that are in the neighborhood of values thought possible for eruption.

There are significant simplifying assumptions that we have built into our mechanical models of the volcanic source. Thermomechanical models that account for lateral heterogeneity^47^ or the effects of a viscoelastic aureole surrounding the modeled reservoir^48^ can modify the resolved depth, overpressure change, and the spatiotemporal geodetic time series^49^. The effects of viscoelastic models can either over- or under-predict surface deformation, compared to the analytical elastic solution. Compared to pressure change elastic solutions, viscoelastic models result in higher deformation, implying that the true pressure change required to fit the observed surface displacements is lower^13^. Whereas, compared to volume change analytical solutions, the viscoelastic models under-predict the deformation, implying that the actual volume change required is greater^13^. Thus, for our CDM source model for volume change rate, our estimate is more likely to underestimate the true value.

For elastic analytical half-space inversions, as in our study, absolute source dimensions may be poorly resolved. However, this primarily affects the over-pressure change in the system, with larger source dimensions resulting in lower pressure changes for a given volume change. Thus, in the case of our Domuyo source model, we estimate a pressure change, *ΔP*, less than 0.5 MPa/year, which would imply a minimum of 20 years of inflation at this rate to reach the 10–50 MPa driving pressure considered necessary to propagate silicic magma to the surface^50^. More robust than pressure change is volume change, which is relatively insensitive to the details of the source dimensions, although for a given source shape it does depend on the source depth—a deeper source requiring greater volume change to produce the same maximum uplift. This is why, for example, our source depth for Domuyo (6.5 km) yields an equal or greater volume change rate compared to Laguna del Maule, whose source is shallower^15^ (4.5 km), despite the latter’s higher uplift rate.

### The nature of the Domuyo volcanic system

**Figure 5:**
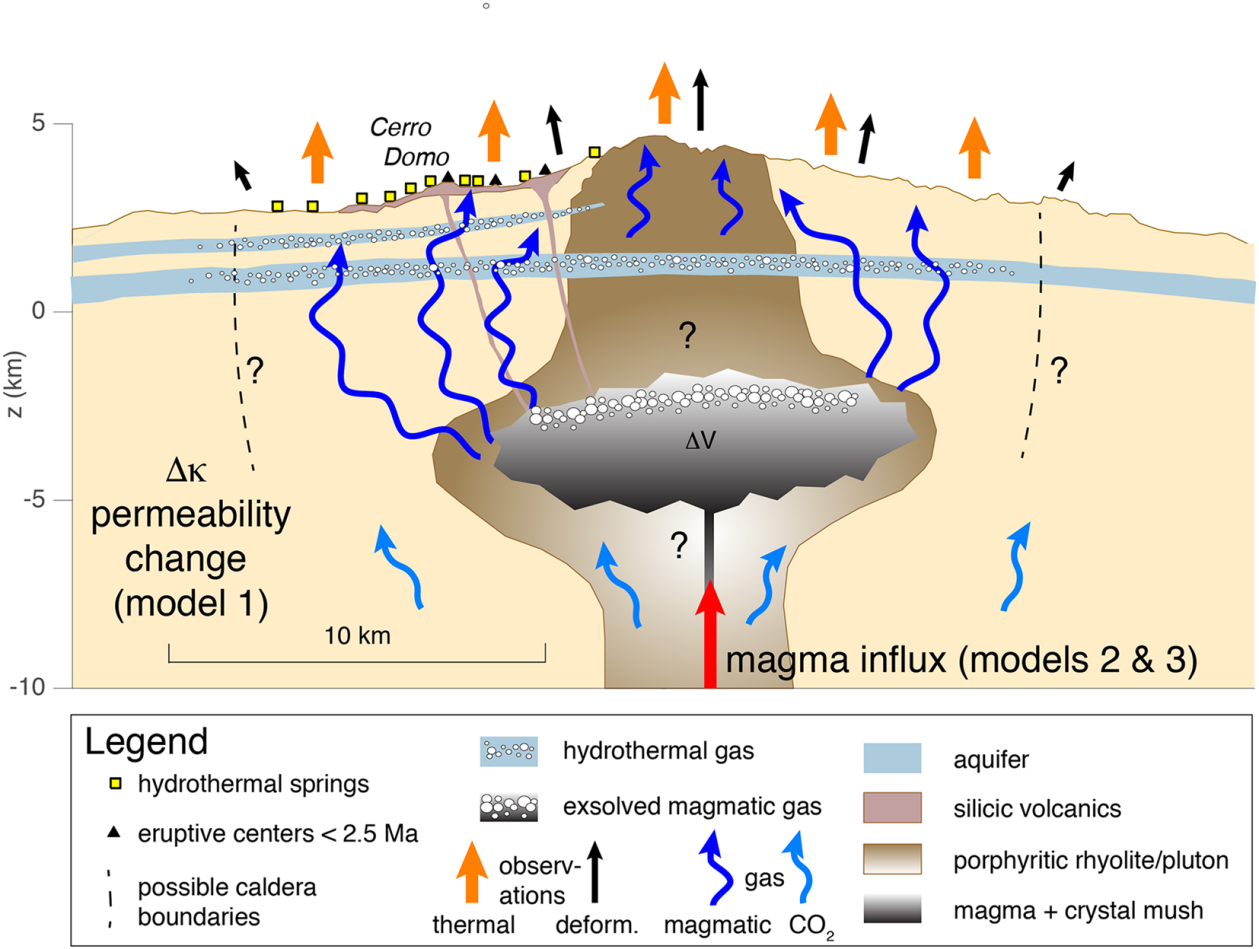
Conceptual model for the Domuyo volcanic system. View from the SE is parallel the long-axis of the modeled ellipsoidal source. Three potential models are based on the concept of magmatic gas percolation between the magma reservoir and the surface, resulting in surface thermal and deformation time series. Model 1 does not require short-term magma influx (red arrow in deep conduit), but derives from passive magmatic degassing from the reservoir ~ 6.5 km below the mean InSAR data surface elevation of 3 km, with shallow change in crust permeability (*κ*) restricting gas, and therefore thermal surface flux, causing gases to build up in the reservoir causing it to inflate at some delay. Model 2 derives from deep (~ 30 km) CO_2_ exsolution from rising magma, resulting in warming leading inflation by more than 5 years. In Model 3, deformation leads heating as magma influx into the system (red arrow) causes reservoir inflation and exsolution of magmatic gas; this results in the transport of magmatic gas towards the surface and interaction with hydrothermal systems, resulting in delayed surface warming.

More or less regular magma injections into the reservoir is also consistent with the current understanding of Domuyo and with interpretations at other silicic magmatic systems (Fig. 5). Structurally, the primary edifice of Domuyo is considered an intrusive dome emplaced in an anticline that folds Permian to Upper Cretaceous marine deposits^51^. The central intruded dome is a high-potassium rhyolite with a porphyric to granophyric texture dated at 2.5 Ma and silicic pyroclastic deposits and lava flows^51^, with Quaternary activity including a 0.72 ± 0.10 Ma basaltic-andesitic to rhyolitic flow^26^, whose most recent activity is a rhyolitic dome (Cerro Domo) dated at 0.11 ± 0.02 Ma (Fig. 1).

A new analysis of gravity, magnetic, and InSAR data from Sentinel-1 only (during the current inflation phase) describes the state of the system^30^. Gravity data suggests that the geodetically inferred 7 km deep source could be fed by a volatile-rich magma^30^. This interpretation is consistent with our second and third models with surface warming driven by an influx of new magma into the shallow (6–7 km depth) reservoir and subsequent gas percolation to the surface and interaction with hydrothermal systems.

Our analysis of surface deformation and surface warming over the last two decades shows clear signs of unrest compatible with Domuyo being an active volcanic system. In particular, the InSAR time series shows an elliptical deformation pattern that projects into the radar LOS for ascending and descending tracks consistent with an upper crustal source. This is confirmed by our solutions for an ellipsoidal source. A sub-horizontal tabular source at ~ 6.5 km depth beneath the surface is deeper than considered possible in granitic crust^52^, is also deeper than the proposed hydrothermal source depths^29^, and suggests the location of the body supplying magmatic gases to the hydrothermal system^27,29^. Hence, the location of our modeled deformation source suggests a long-lived reservoir that is centered beneath the central intrusive dome of Domuyo that we interpret as the likely source for the most recent extrusive eruptions at Cerro Domo and other similar eruptive centers. The aureole of positive dilatation beyond the modeled source is expected to favor gas percolation in the direction of the hydrothermal system, in addition to providing pathways back toward the volcano summit (Fig. 4), producing edifice-scale warming. The surface warming time series shows a thermal variability of the edifice of Domuyo similar to that found at other active volcanoes featuring recent magmatic and gas-driven eruptions^7^, with current thermal emissions at Domuyo on the same order as those observed prior to the last two hydrothermal explosions in 2007 and 2012.

Our findings for Domuyo are relevant to long-term build-up of magma at depth, as it relates to the growth of shallow reservoirs and possible future eruption^53,54^, versus long-term trapping of melt in the crust. This includes, for example, the formation of plutons^55^ and the remobilization of melt within mush and plutons as part of transcrustal magma reservoirs^56,57^. In the case of Domuyo, there is still a great lack of knowledge regarding its geologic history and its present state, although inclusion of gravity observations suggests an influx of melt into the shallow crustal reservoir^30^. The long repose time (~ 100 ka) is not exceptional for large silicic magmatic systems^58^, composed of complex volumes of melt and mush^59,60^. For example, the current activity, if due to magma influx, might represent an incremental injection of melt into a stacked sill complex^54^ that for a longer-lived (> 1 Ma) system such as Domuyo, with intrusion rates on the order of 0.001 km^3^ year^−1^, could build reservoirs capable of large eruptions^61^. The current rate would likely have to be maintained over hundreds of thousands of years in order to approach super-eruption size^62^, highly unlikely to occur soon given the recent deflation preceding the current high influx rate. The current influx rate and modeled source volume (on order 100 km^3^) might be capable of a significant eruption, although remobilization of larger volumes cannot be ruled out^60^. Certainly, we lack sufficient information about the system to develop dynamical models capable of making quantitative, statistically based, probability estimates of future system dynamical behavior.

While the residence times for silicic volcanic systems may be long, remobilization and the run-up to large eruptions may be rapid, with the final batch of mafic recharge occurring over a matter of weeks in the case of the 1912 Katmai, Alaska eruption^63^. However, in general, there is evidence to suggest that the longer the repose time the longer the run-up to eruption is likely to be^64^. To unravel the complexity of a system and forecast its future behavior, as well as to understand whether a new intrusion of magma represents a long process of system evolution or resurgence, requires a vast improvement in our understanding of the state of a given system and integration of diverse time series to track changes in system parameters.

We now have intriguing evidence that Domuyo is a system that is producing deformation and warming signals that are significant and, more importantly, that are comparable to other active volcanoes. How long this system could sustain the current inflation and warming rates, and by inference, build up in pressure in the reservoir before an eruption might begin, is yet to be determined. However, it is important to highlight that: (i) The current inflation phase was preceded by 3-to-5-years of deflation, thus suggesting that we are detecting the regular behavior of this volcano (similar to other calderas in the world) and that an imminent dike propagation and magmatic eruption is not likely. (ii) The current thermal emissions are close to the values found prior to the previous 2007 and 2012 hydrothermal explosions. Hence, although we cannot anticipate an imminent dike propagation and large magmatic eruption, our analysis suggests that the hydrothermal system beneath Domuyo is close to critical pressure values, and thus small hydrothermal gas explosions might occur relatively soon. The InSAR and thermal time series we present show the importance of long-term (decadal) observations in order to track system changes through cross-comparison of multiple observation types to constrain time varying processes. As we have shown with the InSAR, thermal, and seismicity time series, their combination leads to a more focused physical model for the system, while pointing to additional observations and modeling that are needed to constrain the system and to compare with other silicic volcanoes.

## Conclusions

We have combined, for the first time, InSAR and edifice-scale thermal warming time series that provide new constraints on possible physical mechanisms for unrest at large silicic systems. We applied a cross-correlation method to find the correlation amplitudes and relative time lags of the InSAR and thermal time series, revealing that they can be both positively and negatively correlated, with positive and negative thermal lags. These give rise to three candidate models for the current activity of Domuyo volcano (Fig. 5): (1) time-varying permeability of the shallow crust; (2) deep CO_2_ release from mantle magma ascent; and (3) magma-driven reservoir inflation with time-delayed gas percolation. Although the short duration of the time series and the mechanical model alone do not uniquely distinguish between the three aforementioned candidates, the latter (model 3) appears more consistent with the on-going inflation and warming episodes.

Despite the likely magma injection into the shallow reservoir of Domuyo, the integration of long-term surface deformation and surface warming analysis suggests that the volcano is not likely to erupt magma soon, and that it is currently exhibiting a regular caldera-like behavior, with multiple inflation and deflation episodes. However, the possibility that small hydrothermal explosions may occur soon cannot be rejected since thermal emissions are similar to those observed prior to the 2007 and 2012 events. In general, our combination of edifice-scale thermal and geodetic time series provides a new methodological approach for identifying and discriminating between physical mechanisms of unrest at large silicic systems. This will allow a more comprehensive assessment of the current state of volcanoes, as well as drive observations and model development in order to forecast future behavior.

## Methods

### InSAR processing and time series analysis

We explore surface deformation using synthetic aperture radar (SAR) data from four satellite systems: the Canadian Space Agency (CSA) RADARSAT-2 (RSAT2) C-band (5.6 cm wavelength) sensor; the Japan Aerospace Exploration Agency (JAXA) ALOS PALSAR, and ALOS-2 PALSAR-2 L-band (23.8 cm wavelength) sensors; and the Copernicus Sentinel-1a/b C-band (5.6 cm wavelength) satellites operated by the European Space Agency (ESA). For each sensor, we compute InSAR maps of relative ground surface deformation projected into the radar line-of-sight (LOS) direction. Winter snow cover at Domuyo mostly limits the InSAR season to late spring to early fall (December through April), with the start of the season depending on the amount of snow in the previous winter and its termination depending on the onset of new snow in the following winter. The L-band ALOS and ALOS-2 satellites had, in general, better coherence and a slightly longer season compared to RADARSAT-2 and Sentinel-1′s C-band radars, although differences in bandwidth, imaging modes, and temporal sampling affect the performance for each SAR dataset.

Each sensor’s data were processed into differential interferograms of surface deformation, after removal of Earth curvature and topographic effects, with different processing software. For RSAT2, only descending track data were available, and were processed at the Canada Centre for Remote Sensing using the Gamma processing package^65^. ALOS data were available from both ascending and descending tracks; however, large baseline drift of the ALOS satellite limited the interferograms that could be successfully computed. ALOS data were computed at the Jet Propulsion Laboratory (JPL) using the JPL-Caltech (California Institute of Technology) Repeat Orbit Interferometry Package (ROI_PAC) software version 3.1. ALOS-2 and Sentinel-1 SAR data were processed at JPL using the InSAR Scientific Computing Environment (ISCE) package developed at JPL, Caltech, and Stanford University^66^. For Sentinel-1 processing, we used the Advanced Rapid Imaging and Analysis (ARIA) Project at JPL to compute interferograms. ROI_PAC and ISCE processing used the SRTM 30 m digital elevation model (DEM) to correct for topography. Interferogram unwrapping was performed using the SNAPHU software^67^ implemented in ISCE. Plots of interferograms used in the analysis are given in the Supporting Information (Supporting Figs. 1–6).

Both ALOS-2 and Sentinel-1 satellites provide data at Domuyo beginning in the Austral summer of late 2014 or early 2015 through April 2019. We processed a mix of summer-to-summer interferograms (one year maximum for RADARSAT-2 and Sentinel-1; one to four years for ALOS and ALOS-2) and short-term interferograms within each summer season (Fig. 2, Supplementary Fig. 7). Since Domuyo lies in a relatively dry area, the main challenges to InSAR coherence are residual snow and glaciers (especially during the early part of the summer) and volcano topography, which causes geometric effects (layover) and is prone to topographically correlated tropospheric phase delay residuals in the interferograms. The latter is somewhat mitigated by the high elevation (most of the study area is above 2000 m, with the average data elevation used in the modeling at 2,952 m) and relatively dry atmosphere.

ALOS-2 interferograms from ascending path 34 (A34) and descending paths 129 (D129) and 130 (D130) show elliptical patterns of positive line-of-sight (LOS) displacements. This displacement represents movement of the ground towards the satellite radar, that skew towards the satellite look direction for each path, which is consistent with inflation from a buried source (Fig. 2). Differences in the shapes of the observed LOS elliptical patterns depend on the geometry and depth of the source for each imaging mode and path. Over four years (2015–2019), up to ~ 60 cm LOS displacements are observed.

Sentinel-1 interferograms are consistent with the ALOS-2 results, but due to the shorter wavelength and deformation rates around 12 cm/year, interferograms longer than about one year have spatially dense fringes (2π phase cycles), prohibiting unwrapping without significant errors. The ascending and descending tracks of Sentinel-1 also show a similar skewing of the interferogram patterns toward the radar LOS, in accord with an inflating source at depth (Fig. 2).

One-year interferograms from both ALOS-2 and Sentinel-1 showed similar amounts of inflation in year-to-year interferograms from 2015–2019 (and compared to amounts for 4-year interferograms from ALOS-2), suggesting that the deformation began before either satellite’s mission began. This prompted us to look for satellite data that were acquired prior to 2015. A search of ESA’s ERS and Envisat satellites’ C-band SAR acquisitions found no viable pairs during the mid-1990′s through 2009 interval. This was due to both a lack of data and, when data were available, they either had perpendicular baselines (orbital separations) or dopplers (related to radar pointing direction due to changes in satellite attitude variations) that were too large to produce viable interferograms.

ALOS data were processed for the late 2007—early 2011 time period. Only one interferogram was possible for the D412 (descending) path. For the ascending path (A113), 10 SAR images were processed, with only a few producing viable interferograms due to the ALOS baseline drift in the data. Nonetheless, the one D412 interferogram and two of the A113 interferograms show a relatively weak, yet consistent pattern centered on Domuyo volcano (Fig. 2). However, rather than inflation, they show deflation between early 2008 through early 2011 at a lower rate than the post 2014 inflation.

This left RADARSAT-2 to potentially fill the temporal gap between gentle deflation before 2011 and stronger inflation since late 2014. RADARSAT-2 data were available from two acquisition modes: F0W2, a descending track with data from March 2013 to April 2015; and XF0W2, also a descending track, with data from September 2015—mid-2017. The latter track confirms the ALOS-2 and Sentinel-1 observations; but unfortunately, most of the data are from the winter time, so no significant information was provided by the XF0W2 data set. In contrast, the F0W2 data do span the critical interval showing inflation onset (see Fig. 2; Supplementary Figs. 1–6).

We compute InSAR time series from sets of interconnected interferograms using the GIAnT software package^68^ in the NSBAS mode (Supplementary Fig. 7). Output products include both the linear velocity map as well as the full time series. We use the raw (unfiltered) time series, with the displacement uncertainties for each epoch computed from the standard deviation in LOS displacement for a 100-sample set of pixels centered on the point of interest. In Supplementary Fig. 7, we plot the linear velocity map and the InSAR time series for a pixel in the area of peak deformation for each of the ALOS-2, Sentinel-1, RADARSAT-2, and ALOS time series.

From these time series, we see from RSAT2 data that there was no resolvable deformation between 2013 and early 2014. From June 2014 to September 2014, there was an abrupt onset of inflation (within the temporal sampling of the data). We temporally connect the RSAT2 time series to the roughly linear inflation that has continued into 2019 (Fig. 3a) by manually adjusting the time series overlaps. Despite this approach being imprecise because we use different satellites differing in look directions, incidence angles, and points selected for the time series plot, it is suitable for showing the relative deformation behavior at first order. If one were to implement time-variable source modeling of the longer time series, then velocity shifts of each time series could be formally estimated as part of the Bayesian estimation process. In the case of the ALOS time series, there is no temporal overlap with the RSAT2 time series’ zero deformation in early 2013. Therefore, the plot of the ALOS time series in Fig. 3a is plotted to intersect zero deformation in early 2013, coincident with the start of the RSAT2 time series. This was chosen for our plotting purposes since it appears a shorter period of null time series change is in better agreement with the thermal time series null interval from late-2015 to mid-2017. However, this choice does not affect the interpretation and discussion (see Sect. 2.3, where we compare the implications of whether the ALOS time series reaches zero deformation in early 2011 or early 2013).

### Geodetic source modeling

AlTar was run using 4,096 parallel Markov chain Monte Carlo (MCMC) chains, with 1,000 successive steps in which the prior probability distribution for each parameter becomes a progressively more focused Gaussian distribution from its starting uniform range of values. To constrain our model, we use the ALOS-2 linear velocities from the A034 and D129 time series inversions, using a model-based quad-tree approach^69^ with a 10 × 16 km sill at 7 km depth to down-sample the data and compute the covariance matrix. This produced 562 and 454 data points for the A034 and D129 data, respectively.

The model used in the AlTar inversion is the CDM which represents a finite ellipsoidal volume change source composed of three orthogonal dislocations^32^. The model has ten parameters: three for location (*x, y, z*); three semi-axes lengths (*a, b, c*); three axial rotations (*ω*_*x*_*, ω*_*y*_*, ω*_*z*_), allowing for arbitrary orientation and axes lengths; and uniform opening *u* applied to each dislocation in the model. The CDM can approximate shapes ranging from equidimensional, to pancake-shaped, to cigar-shaped, to pipe-like^32^.

### Seismic data

Seismic data are extracted from the Volcano Observatory of the Southern Andes (OVDAS) database, for the time interval from January 2013 through July 2019. We use the seismic networks placed over Laguna del Maule, Longaví, Nevado de Chillán and Tátara volcanoes, located between 40 to 70 km of distance from the Domuyo area. The horizontal and vertical errors of the seismic locations obtained are less than 3 km, providing a good representation of the local seismicity occurring during recent years close to Domuyo volcanic edifice. A total of 83 seismic events were detected, with *M*_*L*_ higher than 2.0; the minimum magnitude to obtain locations with OVDAS seismic stations. A remarkable aspect is the increase in seismic occurrence and seismic energy between 2016 and 2019, especially in March 2019 when the most energetic earthquake (*M*_*L*_* 4.1*) was recorded (Fig. 3c).

### TIR data processing

We explore the thermal emissions of the flanks of Domuyo volcano using a new algorithm that examines the thermal infrared (TIR) data provided by the MODIS instruments aboard NASA’s Terra and Aqua satellites^7^. In particular, we downloaded (from the NASA Earth Data Search site https://earthdata.nasa.gov/) the Level-1B Radiance product (MOD021KM/MYD021KM) and the Level-1A Geolocation product (MOD03/MYD03) to analyze a more than 17-year record (from 04-July-2002 to 20-August-2019), of day- and night-time scenes, from MODIS Band 31 (10.780–11.280 ${\mu m}$). This gives a total of ~ 3.8 TB of memory, 28,407 scenes, and average sampling rate of ~ 4–5 scenes per day at Domuyo’s latitude, allowing us to update Domuyo’s warming time series from that presented in Girona et al. ^7^ The algorithm computes a statistical metric, the median anomaly ($\overline{\delta T}$), that tracks the long-term (~ years) variations of the median brightness temperature of a volcanic edifice relative to the median brightness temperature of the region surrounding the volcano. The median anomaly $\overline{\delta T}$ is computed following the six main steps summarized below^7^.

First, we choose a target area around Domuyo volcano on the order of $A_{t} \sim 1,400 {\text{km}}^{2}$ (which corresponds to a total of $N \approx 200 - 1,350$ pixels, depending on the observation angle of each scene) and an auxiliary area of $A_{aux} \sim 800 {\text{km}}^{2}$, embedded within $A_{t}$, that covers the edifice and includes the hydrothermal field at the southwest flank of the volcano^27,29^. Second, we calculate the median spectral radiance of the $M = 11$ pixels with lowest spectral radiance ($L_{c,M}$), as well as the median spectral radiance of the $K = N - 101$ pixels with largest spectral radiance ($L_{h,K}$), of the target area $A_{t}$. In the absence of clouds, the pixels with the lowest spectral radiance and the pixel with median spectral radiance $L_{c,M}$ fall into the subarea $A_{aux}$, whereas the pixels with largest spectral radiance $L_{h,K}$ fall outside the subarea $A_{aux}$; this is so because the highest levels of the volcano are colder due to altitude. If the pixel with median spectral radiance $L_{c,M}$ does not fall into the subarea $A_{aux}$, and/or the pixel with median spectral radiance fall into the subarea $A_{aux}$, the scene is discarded from the analysis to reduce cloud-related noise. This yields 30% of scenes discarded and a total of 19,982 useful scenes for the ~ 17-year period under study. Third, $L_{c,M}$ and $L_{h,K}$ are converted to brightness temperature ($T_{c,M}$ and $T_{h,K}$, respectively) using Planck’s function and the central wavelength of Band 31, and then we calculate the difference $\Delta T_{M,K} = T_{c,M} - T_{h,K}$. Fourth, we calculate the daily median of the differences of brightness temperature, $\overline{\Delta T}_{M,K}$; the resulting time series ($\overline{\Delta T}_{M,K} \left( t \right)$, where $t$ is time) displays as one data point per day with a seasonal component, noise, and 17% gaps (due to the scenes discarded in step 2 of the algorithm) that are filled through linear interpolation. Fifth, $\overline{\Delta T}_{M,K} \left( t \right)$ is filtered through an iterative technique based on wavelet and moving median filters that efficiently removes seasonality and noise. This yields the median anomaly or $\overline{\delta T}$, which is negative (because $T_{c,M} < T_{h,K}$), has units of temperature, and captures long-term ($> 1$ year) variations of the brightness temperature of the coldest parts of volcanic edifices relative to the region surrounding the volcano. Sixth, the uncertainty of the $\overline{\delta T}$ time series is assessed through a combination of bootstrapping and Monte Carlo techniques. For simplicity, $\overline{\delta T}$ (with its uncertainty) is rescaled, so it is always $\ge 0$.

### Cross-correlation of deformation and warming time series

We assess the possible link between deformation and warming through a cross-correlation analysis over the 10-year period when both time series are available (from early 2008 to mid-2018). We linearly interpolate the deformation to one data value per day, mimicking the effective sampling rate of the surface warming time series. In addition, we perform the cross-correlation analysis for two cases: (1) when assuming that ALOS InSAR time series reaches zero deformation (subsidence stops) in early 2013, coincident with the start of the RSAT2 data (red curve in Fig. 3b); and (2) when assuming that ALOS reaches zero at the end of the ALOS data in early 2011 (blue curve in Fig. 3b; see Supplementary Fig. 12 for a more thorough comparison of time series shifts). Both cases yield similar results: cross-correlation analysis shows a high degree of similarity between both time series at three specific time-shifts (Fig. 3b). It is worth highlighting that both the positive and negative correlations are highly significant, with the positives being smaller (in absolute value) than the negative (< 60% vs. 100% of normalized cross-correlation).

### Porous flow-induced time delay

We assume that a good proxy for the timescale of heat transport to the surface is the timescale of permeable flow of gas from the reservoir to the hydrothermal system (justified because the remaining processes occur at shallower levels beneath the surface). In such a case, we can estimate the time lag ($\tau$) between surface deformation and warming in terms of the mean seepage velocity ($\overline{v\left( z \right)}$) of gas through the crust:

1 $$\tau = \frac{L}{{\overline{v\left( z \right)} }} = \frac{L}{{\frac{1}{L}\mathop \smallint \nolimits_{z = 0}^{z = L} v\left( z \right)dz}} = \frac{{L^{2} }}{{\mathop \smallint \nolimits_{z = 0}^{z = L} v\left( z \right)dz}},$$

where $L$ is the depth to the top of the magma source as measured from the hydrothermal system (which roughly equals the magma source depth because hydrothermal systems are typically much shallower), $v\left( z \right)$ is the local seepage velocity through the crust, and $z$ is the distance measured positive from the top of the magma reservoir. The seepage velocity can be estimated from the following constitutive equation (Darcy’s law):

2 $$v\left( z \right) = - \frac{\kappa }{\mu \varphi }\frac{{{\text{d}}p\left( z \right)}}{{{\text{dz}}}}$$

where $\kappa$ and $\varphi$ are the effective permeability and porosity of the crust, respectively; $\mu$ is the gas viscosity; and $p\left( z \right)$ is the pressure with depth (measured positive upwards) in the crust. Replacing Eq. (2) into the integral of Eq. (1) yields:

3 $$\tau = - \frac{{L^{2} }}{{\frac{\kappa }{\mu \varphi }\mathop \smallint \nolimits_{z = 0}^{z = L} \frac{{{\text{d}}p\left( z \right)}}{{{\text{dz}}}}dz}} = \frac{{\mu \varphi L^{2} }}{{\kappa \left( {P_{r} - P_{c} } \right)}}$$

where $P_{r}$ is the reservoir pressure, $P_{c}$ is the pressure in the hydrothermal system near the surface, and we can approximate $P_{r} - P_{c} \approx \rho gL$, where $\rho gL$ is the lithostatic pressure at reservoir depths, $\rho$ is the crust density, and $g$ is gravity. Substituting into Eq. (3), we obtain the diffusion time, or time required for the gas to ascend near the surface from a magma reservoir:

4 $$\tau = \frac{\mu \varphi L}{{\kappa \rho g}}.$$

Equation (4) reveals that, under the assumptions considered, the time delay between deformation and surface warming at Domuyo is directly proportional to the crust porosity and reservoir depth, and inversely proportional to crust permeability and density. This equation allows us to constrain the effective permeability of the crust from our geodetic and thermal infrared analysis. In particular, using realistic values of the parameters, i.e., $\mu = 10^{ - 5} {\text{Pa s}}$, $\varphi = 10^{ - 2} - 10^{ - 4}$, $L = 5,700 {\text{m}}$ (top of the reservoir, as inferred from InSAR analysis), $\rho = 3,000 {\text{kg}}/{\text{m}}^{3}$, $g = 9.8 {\text{m}}/{\text{s}}^{2}$, and $\tau = 2.7 {\text{years}}$ (as inferred from the comparison between InSAR and thermal data), we find an effective permeability in the range $\kappa \approx 10^{ - 18} - 10^{ - 16} {\text{m}}^{2}$. This is consistent with typical estimates of shallow crust permeability in volcanic areas^70^.

## Supplementary information

Supplementary Information.
