# Supplementary material for: The dynamics of large silicic systems from satellite remote sensing observations: the intriguing case of Domuyo volcano, Argentina
Source: Sci Rep. 2020 Jul 15;10:11642. doi: 10.1038/s41598-020-67982-8 (PMC7363862; doi:10.1038/s41598-020-67982-8)
Supplement: Supplementary file 1 — Supplementary Information. [file 41598_2020_67982_MOESM1_ESM.docx]

The dynamics of large silicic systems from satellite rsemote sensing observations: the intriguing case of Domuyo volcano, Argentina

P. Lundgren^1^*, T. Girona^1^, M.G. Bato^1^, V. Realmuto^1^, S. Samsonov^2^, C. Cardona^3^, L. Franco^3^, E. Gurrola^1^, and M. Aivazis^4^

^1^Jet Propulsion Laboratory, California Institute of Technology, Pasadena, CA, USA.

^2^Canada Centre for Mapping and Earth Observation, Natural Resources Canada, Ottawa, Canada.

^3^Observatorio Vulcanológico de los Andes del Sur (OVDAS), Servicio Nacional de Geología y Minería, Temuco, Chile.

^4^Parasim Inc., Pasadena, CA, USA.

*Corresponding author: Paul Lundgren (paul.lundgren@jpl.nasa.gov)

**Supplementary Material**

Supplementary Table 1: Compound Dislocation Model (CDM) AlTar maximum a-posteriori (MAP) solution and parameter standard deviation (2σ) uncertainties derived from the distributions in Supplementary Figure 8. All map coordinates (x, y) are in a local cartesian coordinate system centered at: Longitude -70.43, Latitude -36.63, the approximate summit of Domuyo.

| **Parameter** | **Value** |
| --- | --- |
| *x* (m) | -259 ± 26 |
| *y* (m) | 2016 ± 26 |
| *z* (m) | -6455 ± 80 |
| *a* semi-axis (m) | 5662 ± 42 |
| *b* semi-axis (m) | 3872 ± 66 |
| *c* semi-axis (m) | 685 ± 54 |
| *ω_x_* (degrees) | -3.8 ± 0.56 |
| *ω_y_* (degrees) | 2.7 ± 0.36 |
| *ω_z_* (degrees) | 44.3 ± 0.72 |
| *u* (m/yr) | 0.328 ± 0.0084 |
| Offset ascending LOS velocity data (m/yr) | 0.0019 ± 0.00028 |
| Offset descending LOS velocity data (m/yr) | 0.0110 ± 0.00042 |
| dV (km^3^/yr) | 0.0373 ± 0.0010 |

**
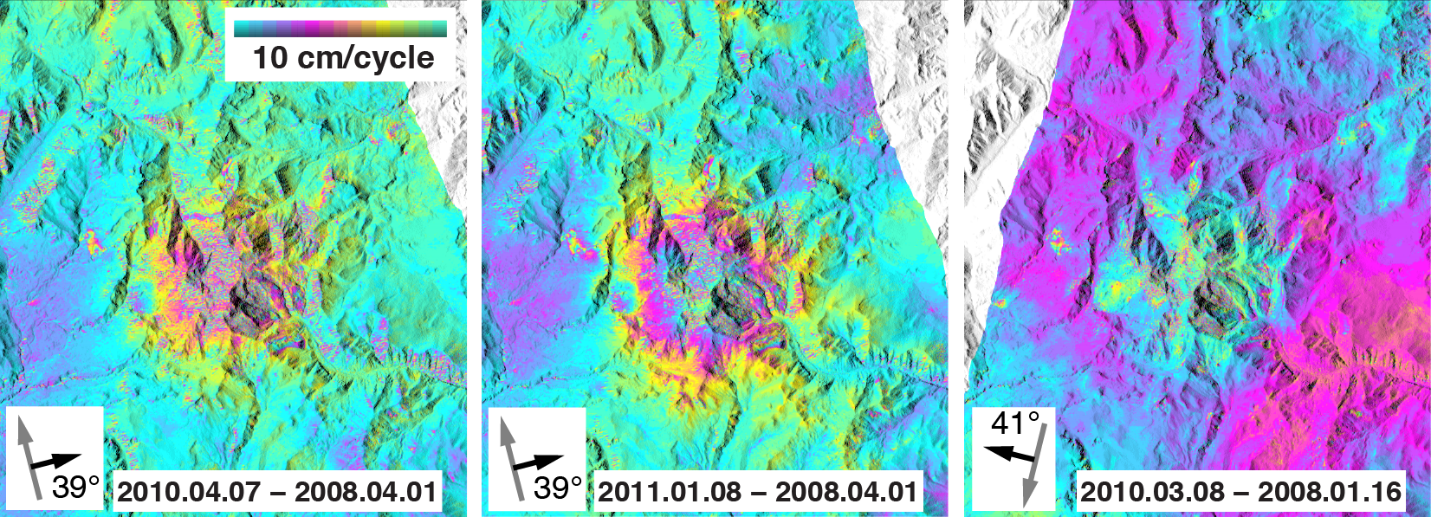
**

Supplementary Figure S1. ALOS SAR interferograms analyzed. The left and center interferograms are for ascending path 113, and used in the time series analyses (shown in Figure S1). The interferogram on the right is from descending path 417 and shows a similar deflation pattern as seen on the left for a similar ~2-year time interval from early 2008 to early 2010. Note that each interferogram is a sub-area of the original interferogram and, therefore, each interferogram shown has a slightly different, non-zero phase value in the far-field, leading to apparent shifts in the background color. This reflects the ‘relative’ LOS displacements (in both space and time) shown by any interferogram.


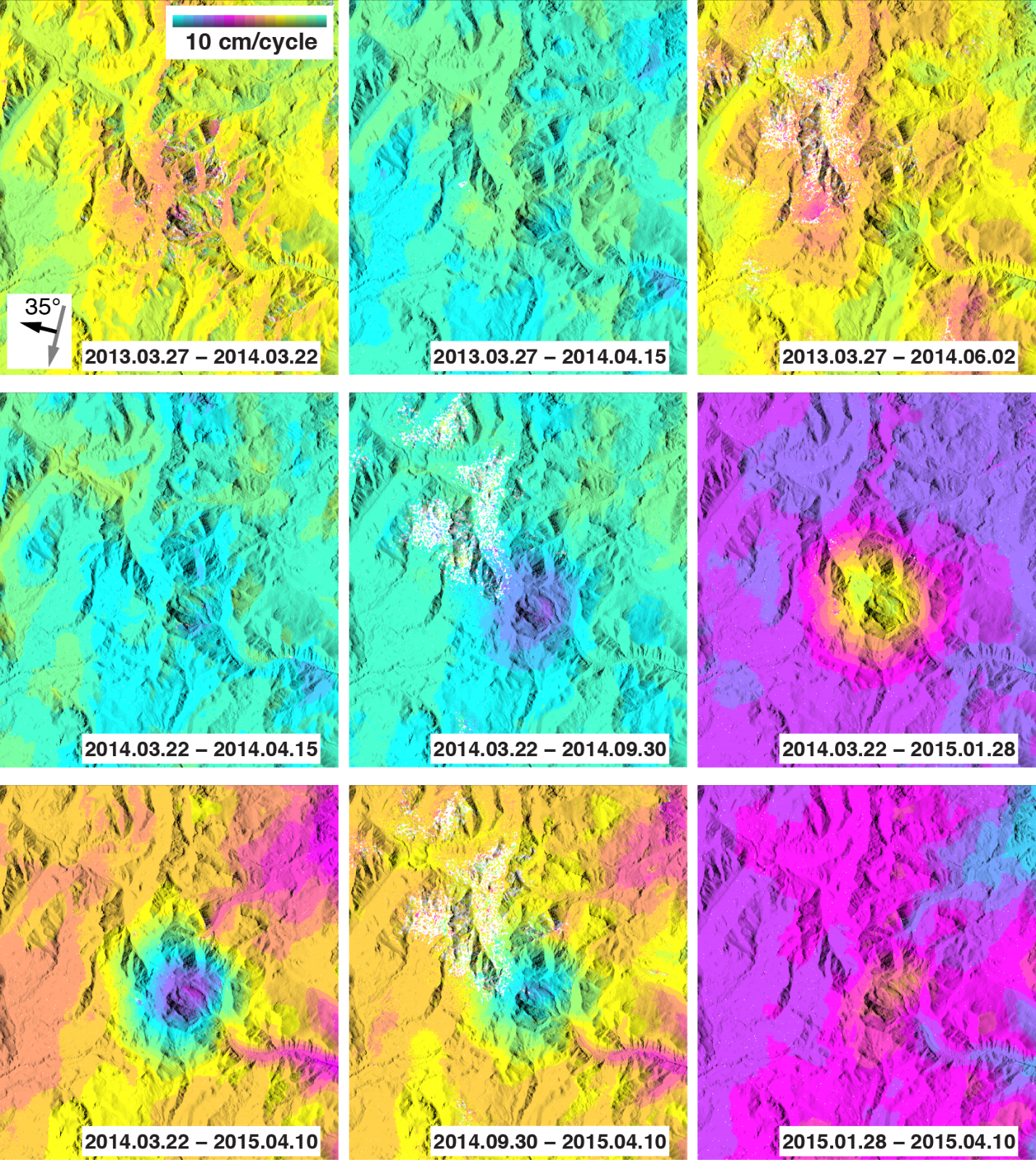


Supplementary Figure 2. RADARSAT-2 SAR interferograms analyzed. All interferograms are from the same descending track in imaging mode F0W2.


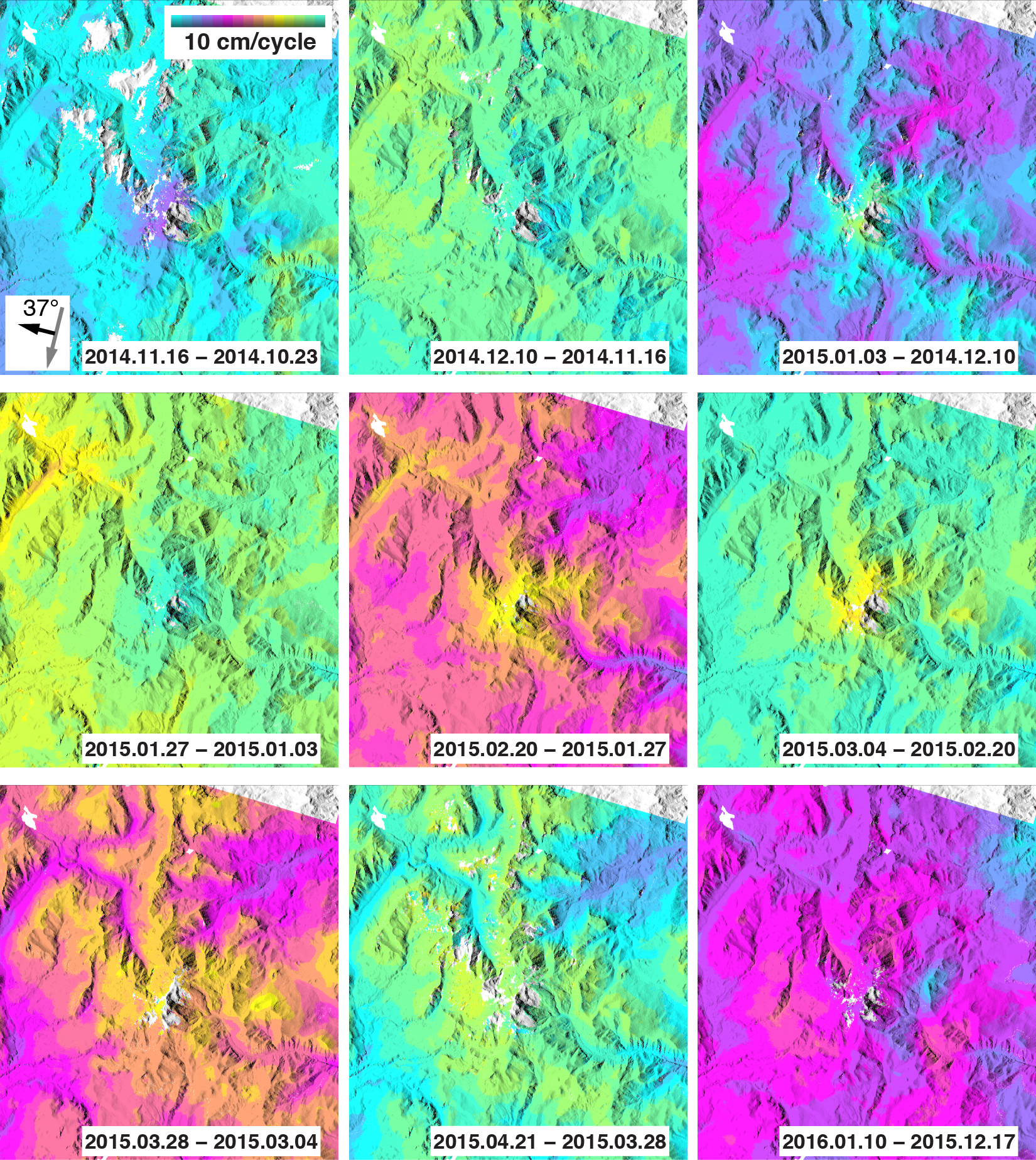


Supplementary Figure 3a. Sentinel-1 SAR interferograms analyzed using the JPL-Caltech ARIA (Advanced Rapid Imaging and Analysis) InSAR processing system. All interferograms are from the same descending track 83.


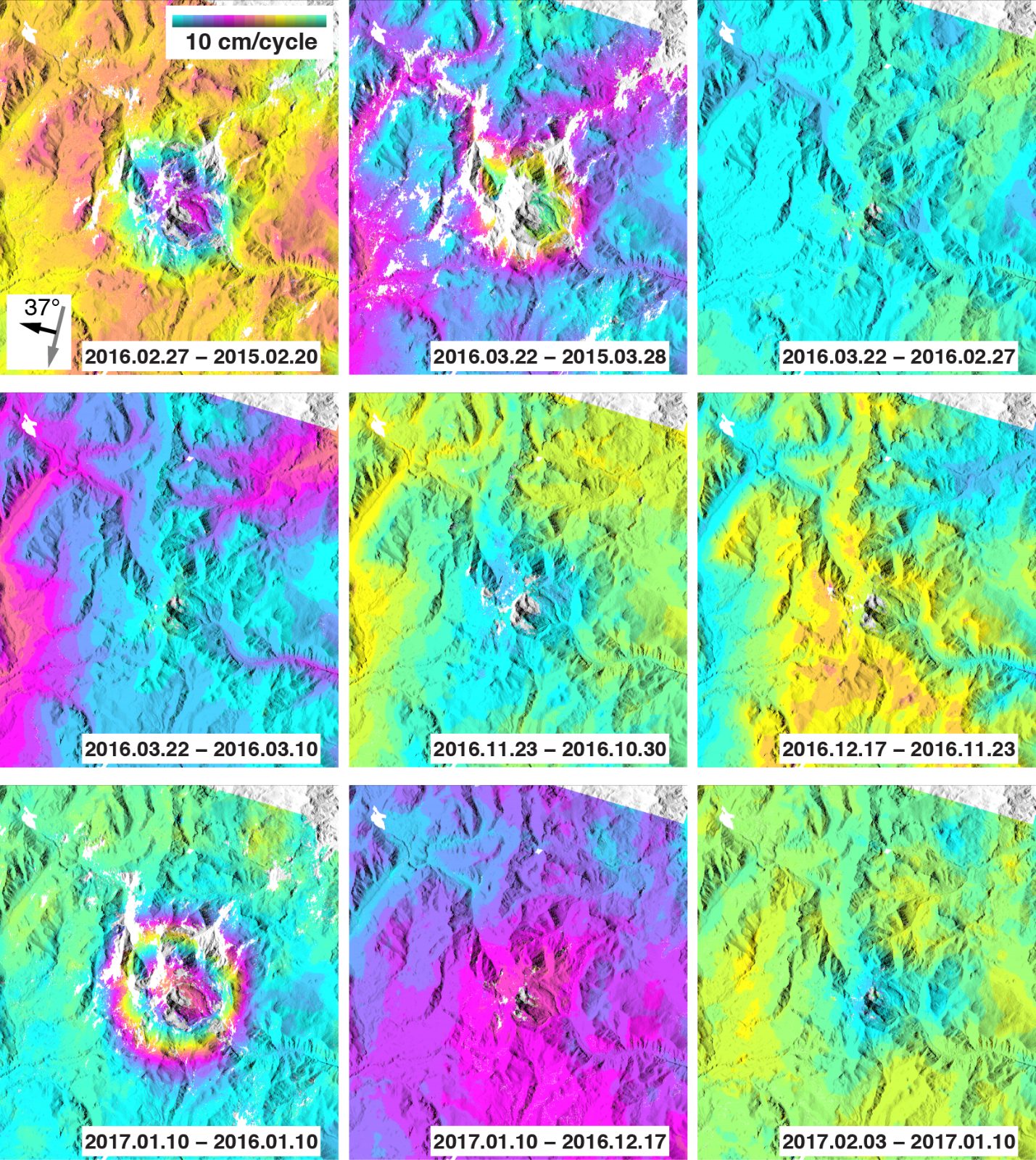


Supplementary Figure 3b. Sentinel-1 SAR interferograms analyzed using the JPL-Caltech ARIA (Advanced Rapid Imaging and Analysis) InSAR processing system. All interferograms are from the same descending track 83.


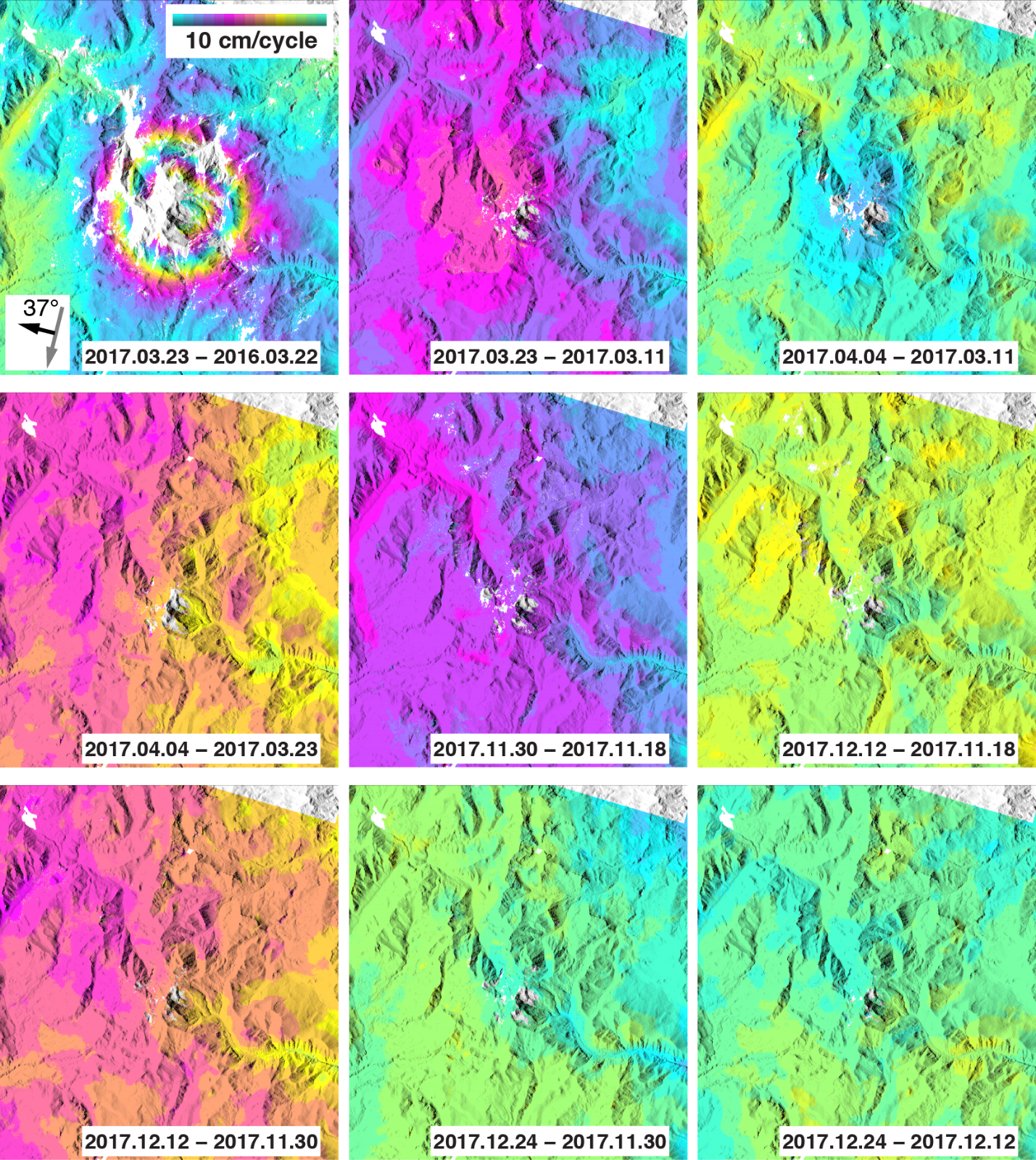


Supplementary Figure 3c. Sentinel-1 SAR interferograms analyzed using the JPL-Caltech ARIA (Advanced Rapid Imaging and Analysis) InSAR processing system. All interferograms are from the same descending track 83.


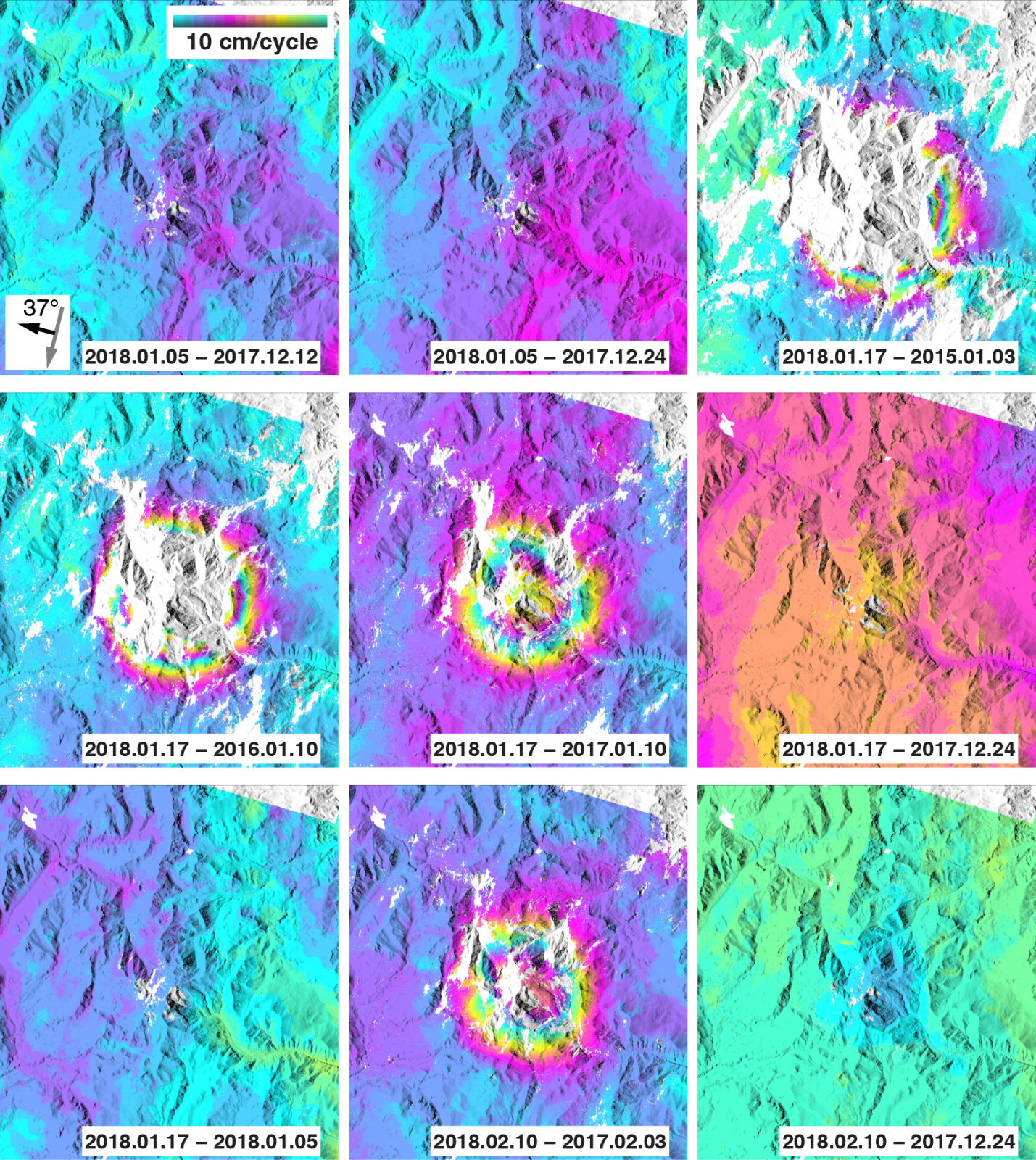


Supplementary Figure 3d. Sentinel-1 SAR interferograms analyzed using the JPL-Caltech ARIA (Advanced Rapid Imaging and Analysis) InSAR processing system. All interferograms are from the same descending track 83.


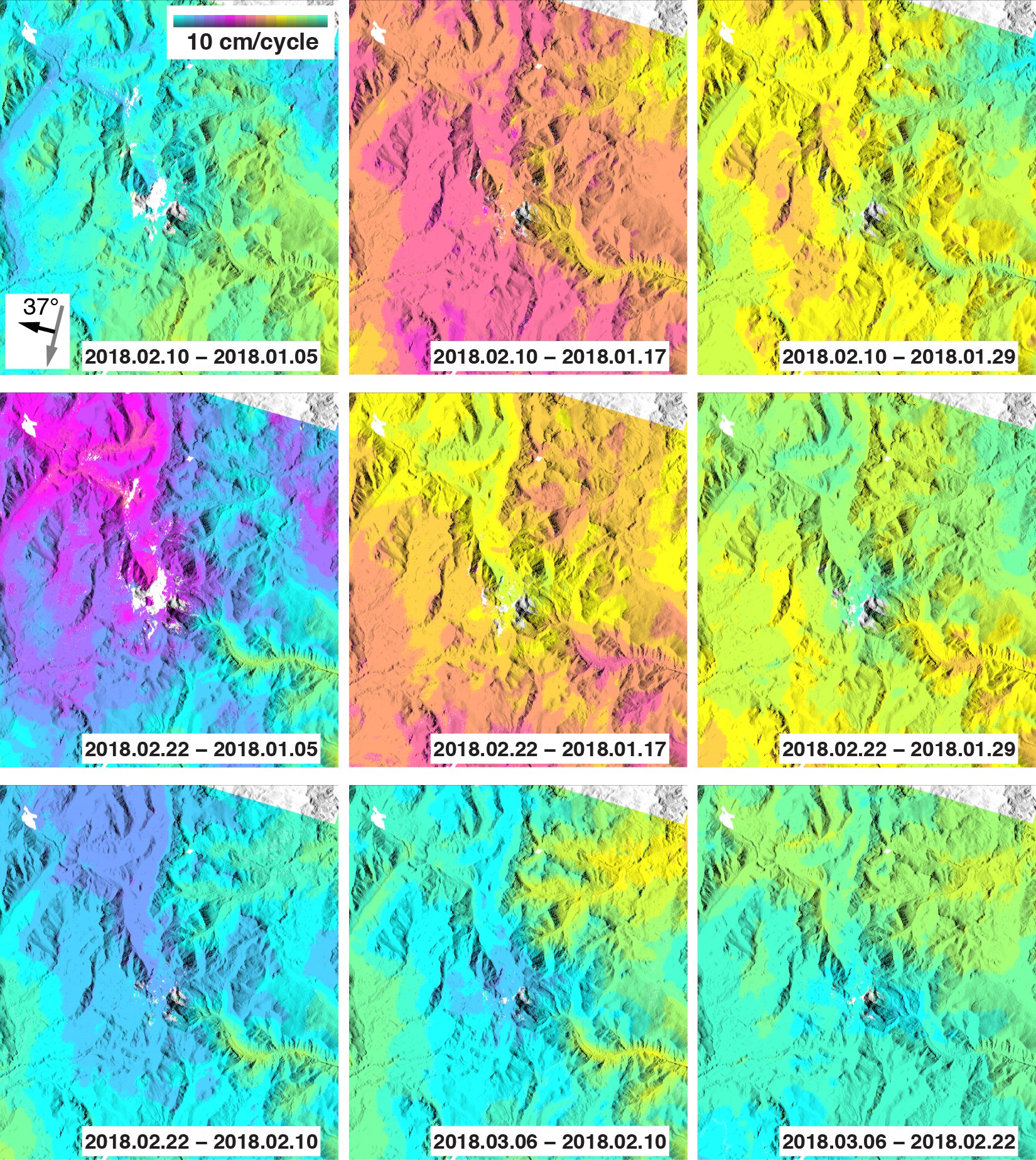


Supplementary Figure 3e. Sentinel-1 SAR interferograms analyzed using the JPL-Caltech ARIA (Advanced Rapid Imaging and Analysis) InSAR processing system. All interferograms are from the same descending track 83.


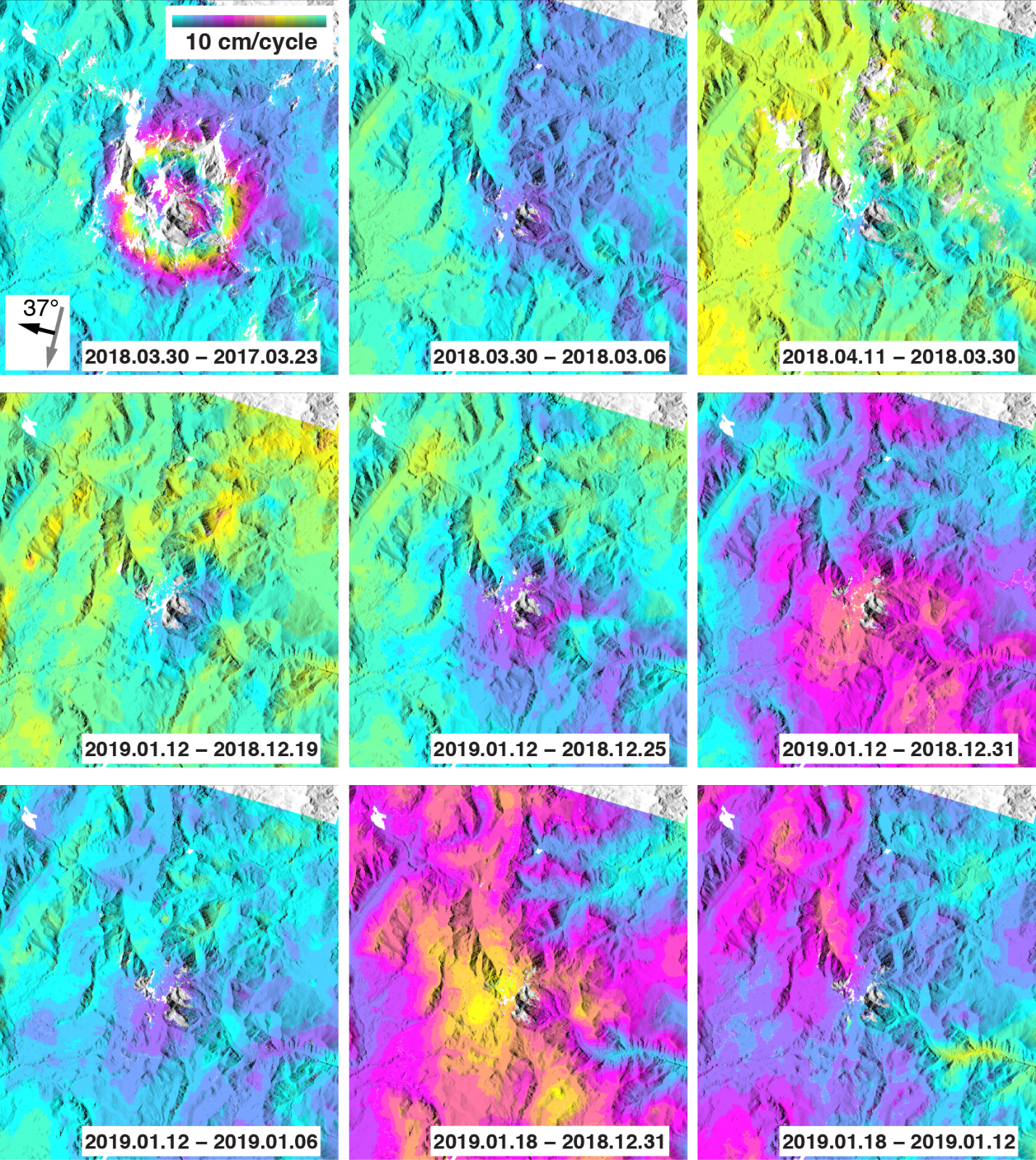


Supplementary Figure 3f. Sentinel-1 SAR interferograms analyzed using the JPL-Caltech ARIA (Advanced Rapid Imaging and Analysis) InSAR processing system. All interferograms are from the same descending track 83.


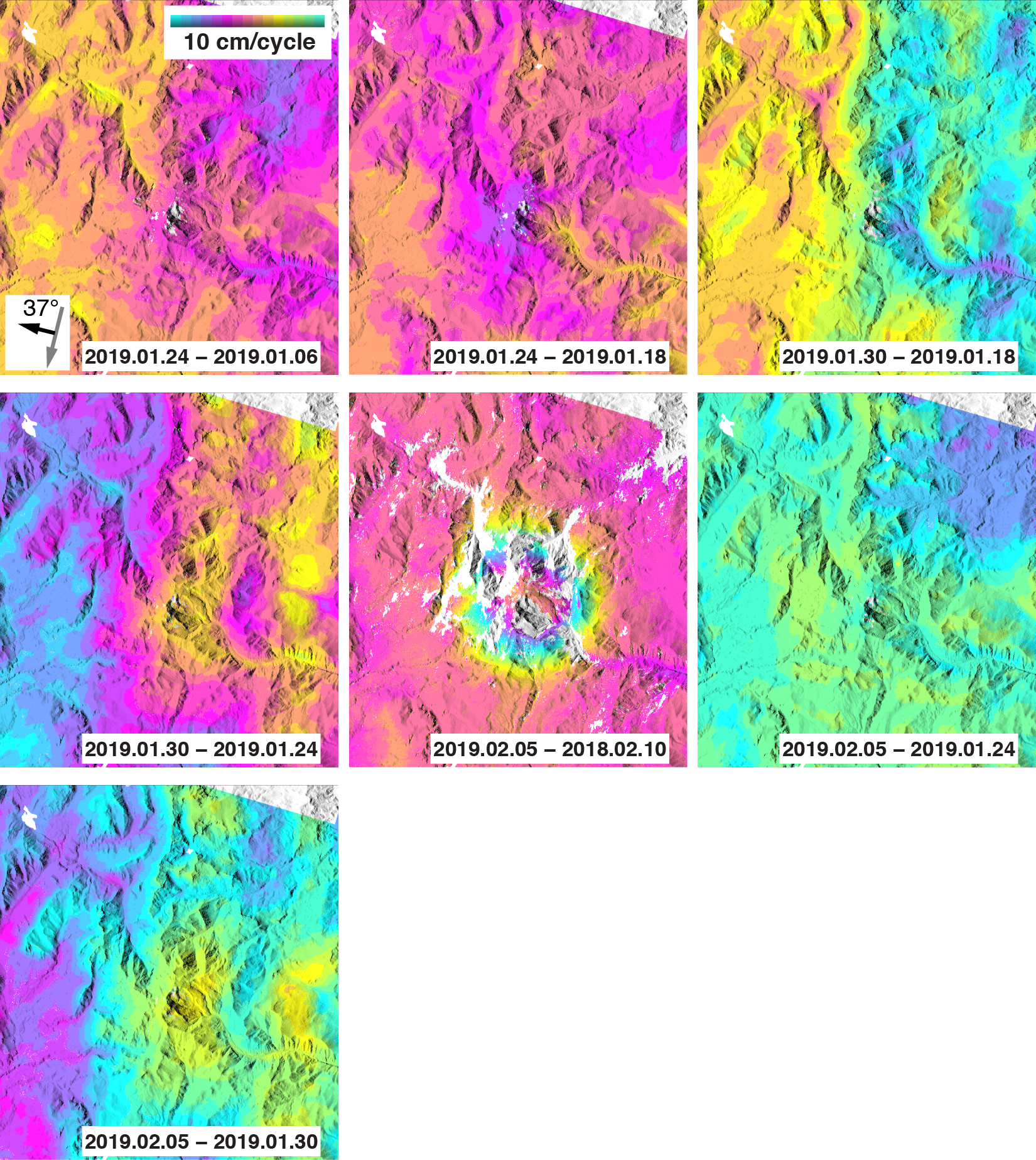


Supplementary Figure 3g. Sentinel-1 SAR interferograms analyzed using the JPL-Caltech ARIA (Advanced Rapid Imaging and Analysis) InSAR processing system. All interferograms are from the same descending track 83.


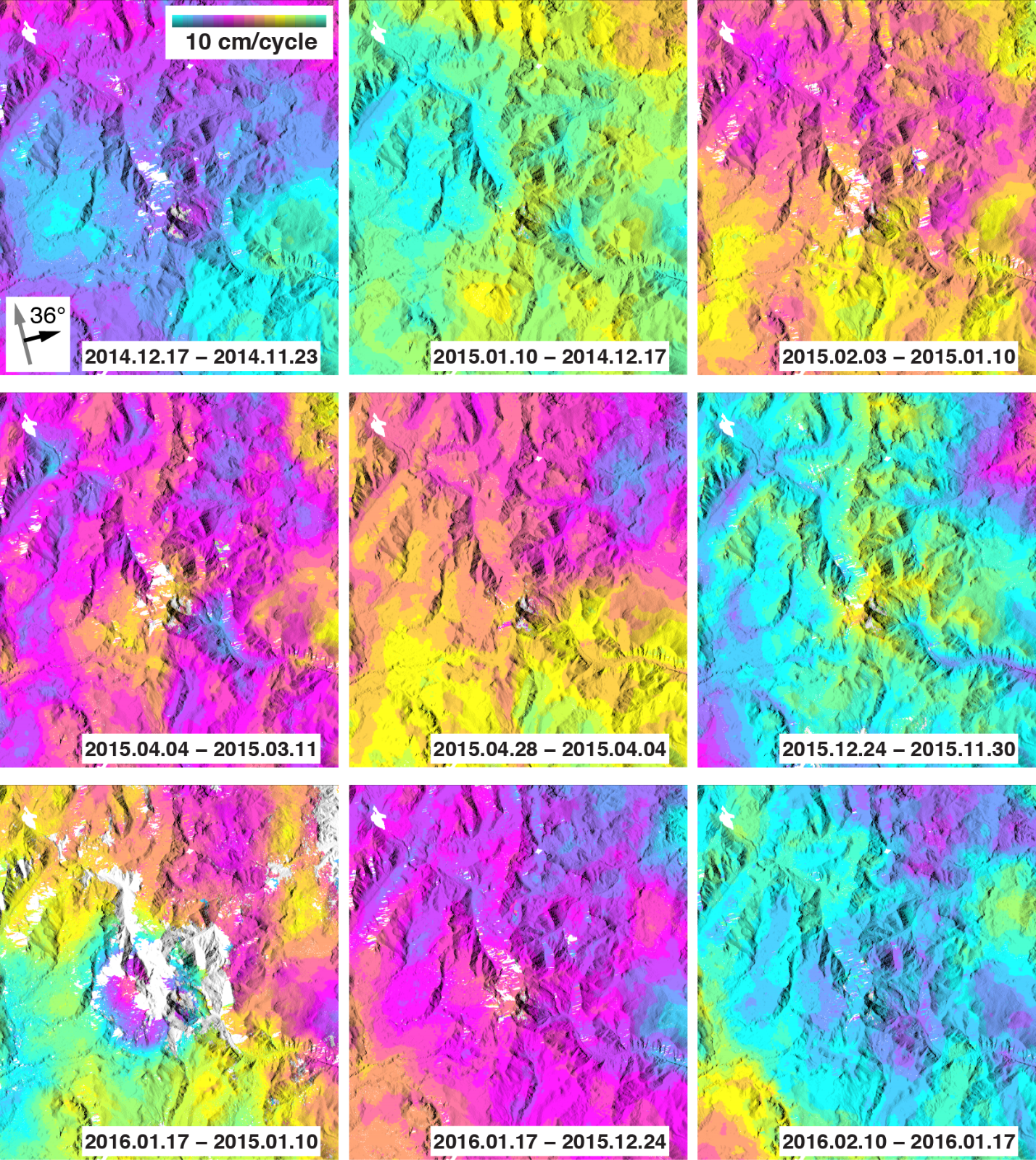


Supplementary Figure 4a. Sentinel-1 SAR interferograms analyzed using the JPL-Caltech ARIA (Advanced Rapid Imaging and Analysis) InSAR processing system. All interferograms are from the same ascending track 18.


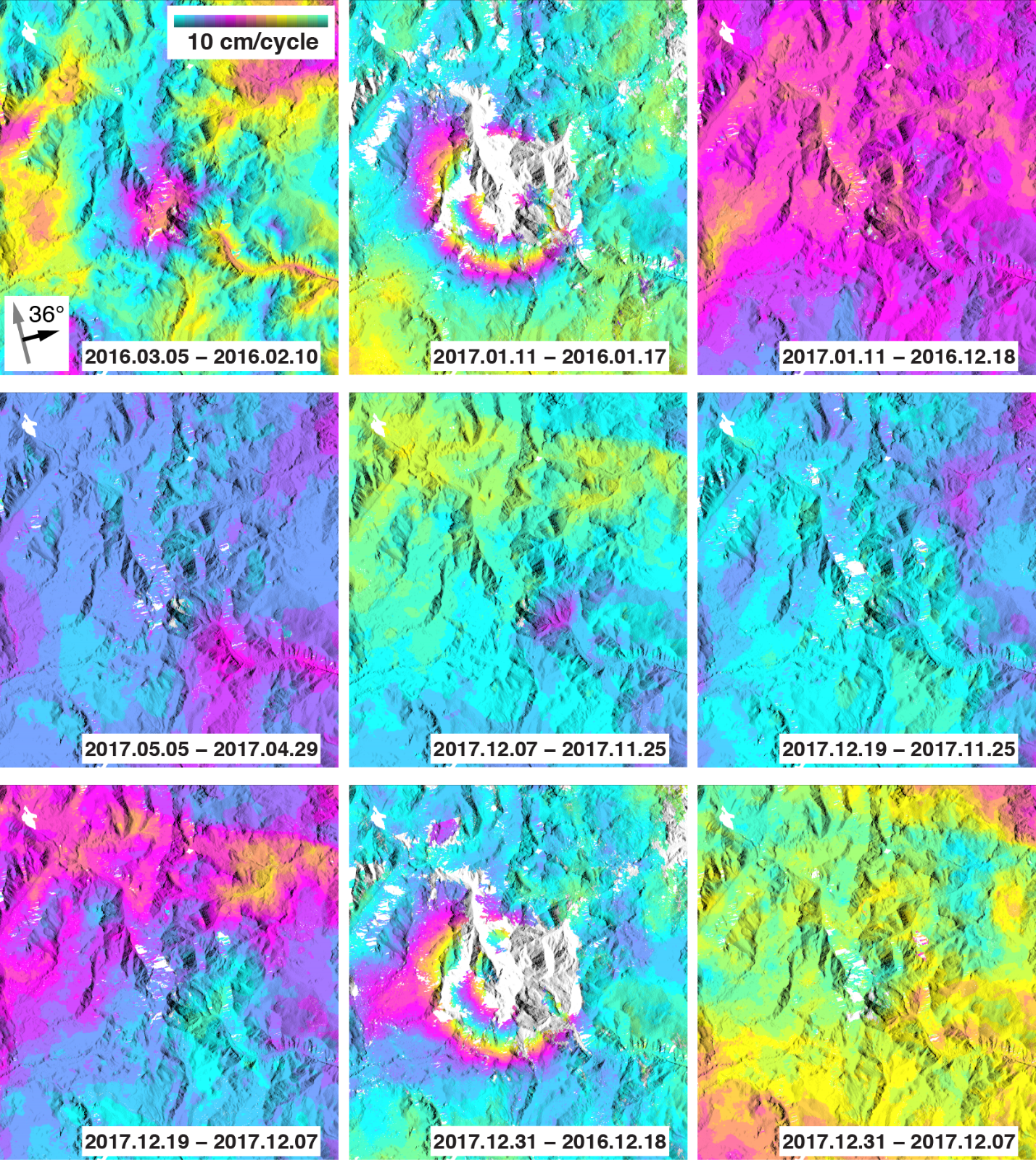


Supplementary Figure 4b. Sentinel-1 SAR interferograms analyzed using the JPL-Caltech ARIA (Advanced Rapid Imaging and Analysis) InSAR processing system. All interferograms are from the same ascending track 18.


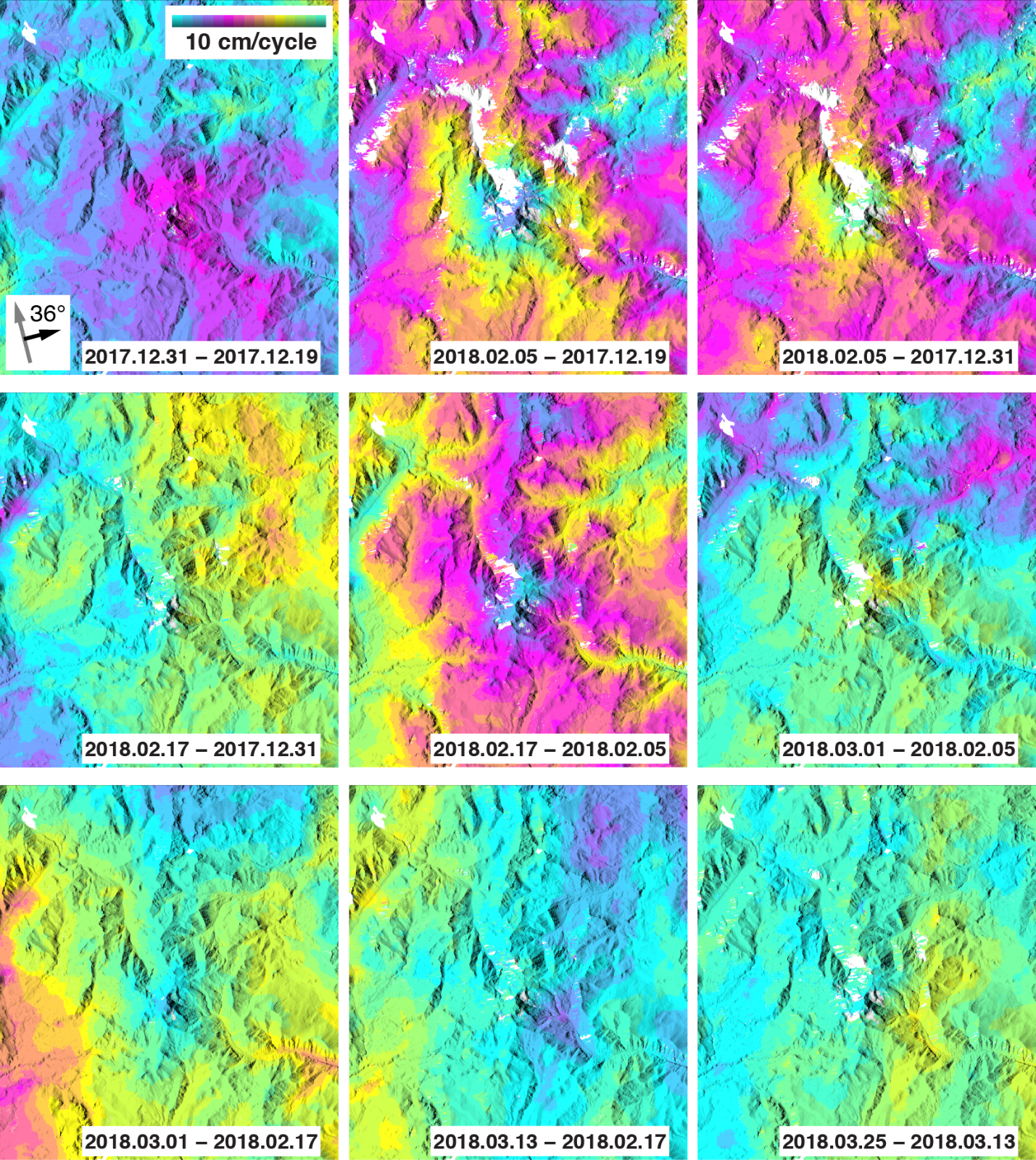


Supplementary Figure 4c. Sentinel-1 SAR interferograms analyzed using the JPL-Caltech ARIA (Advanced Rapid Imaging and Analysis) InSAR processing system. All interferograms are from the same ascending track 18.


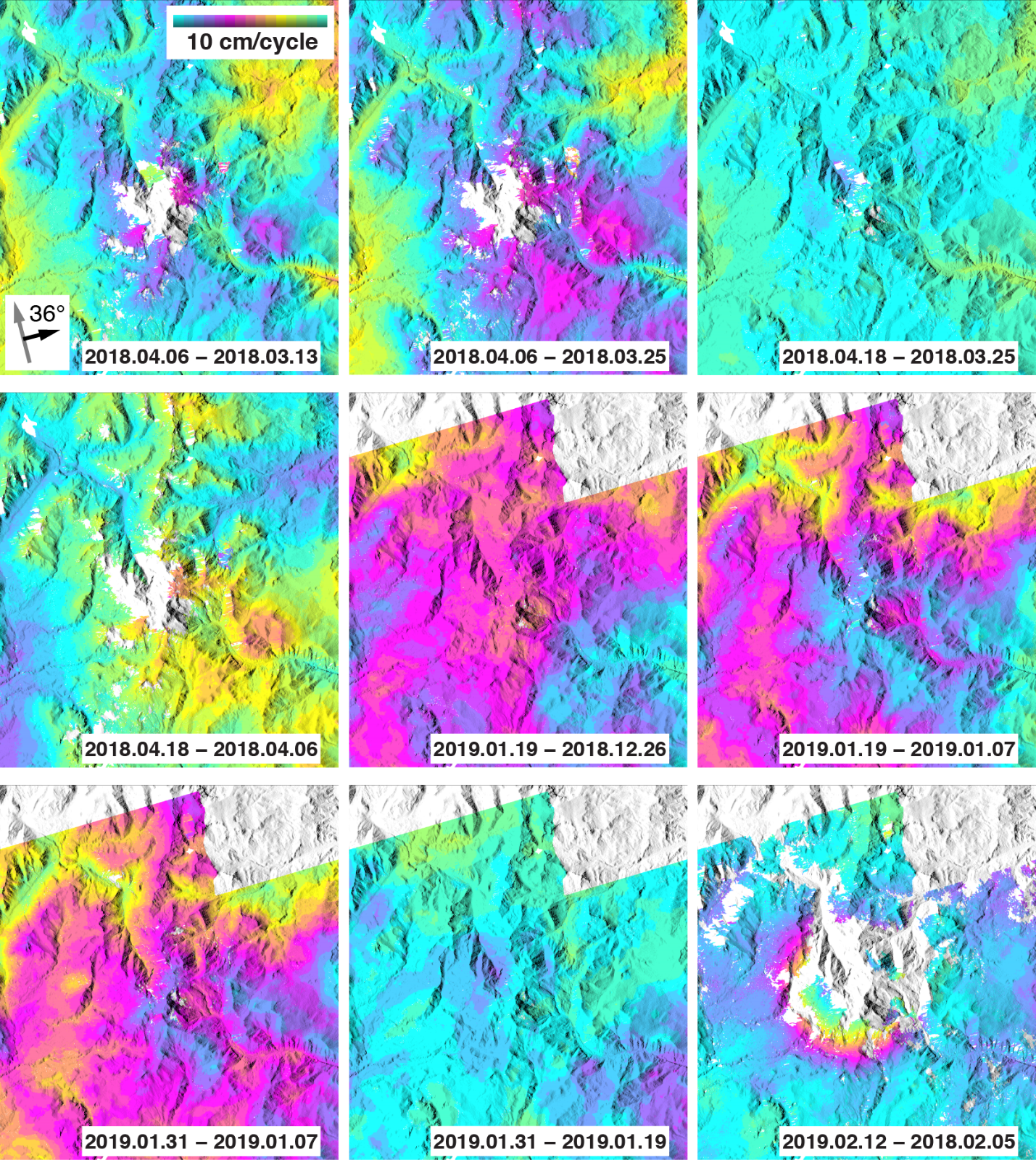


Supplementary Figure 4d. Sentinel-1 SAR interferograms analyzed using the JPL-Caltech ARIA (Advanced Rapid Imaging and Analysis) InSAR processing system. All interferograms are from the same ascending track 18.


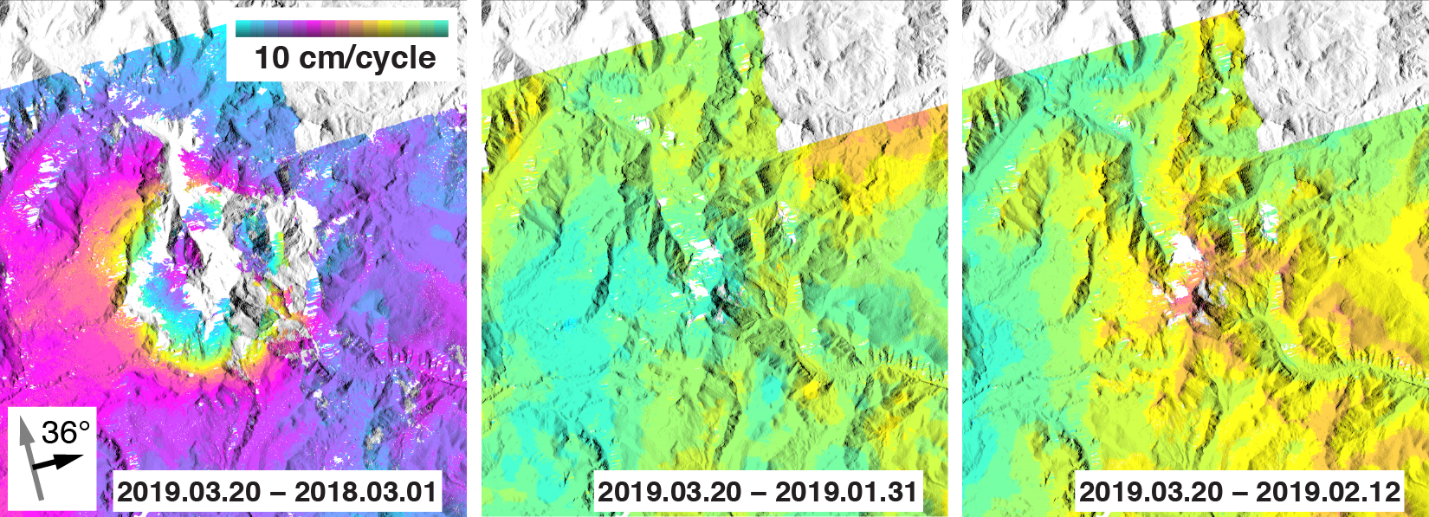


Supplementary Figure 4e. Sentinel-1 SAR interferograms analyzed using the JPL-Caltech ARIA (Advanced Rapid Imaging and Analysis) InSAR processing system. All interferograms are from the same ascending track 18.


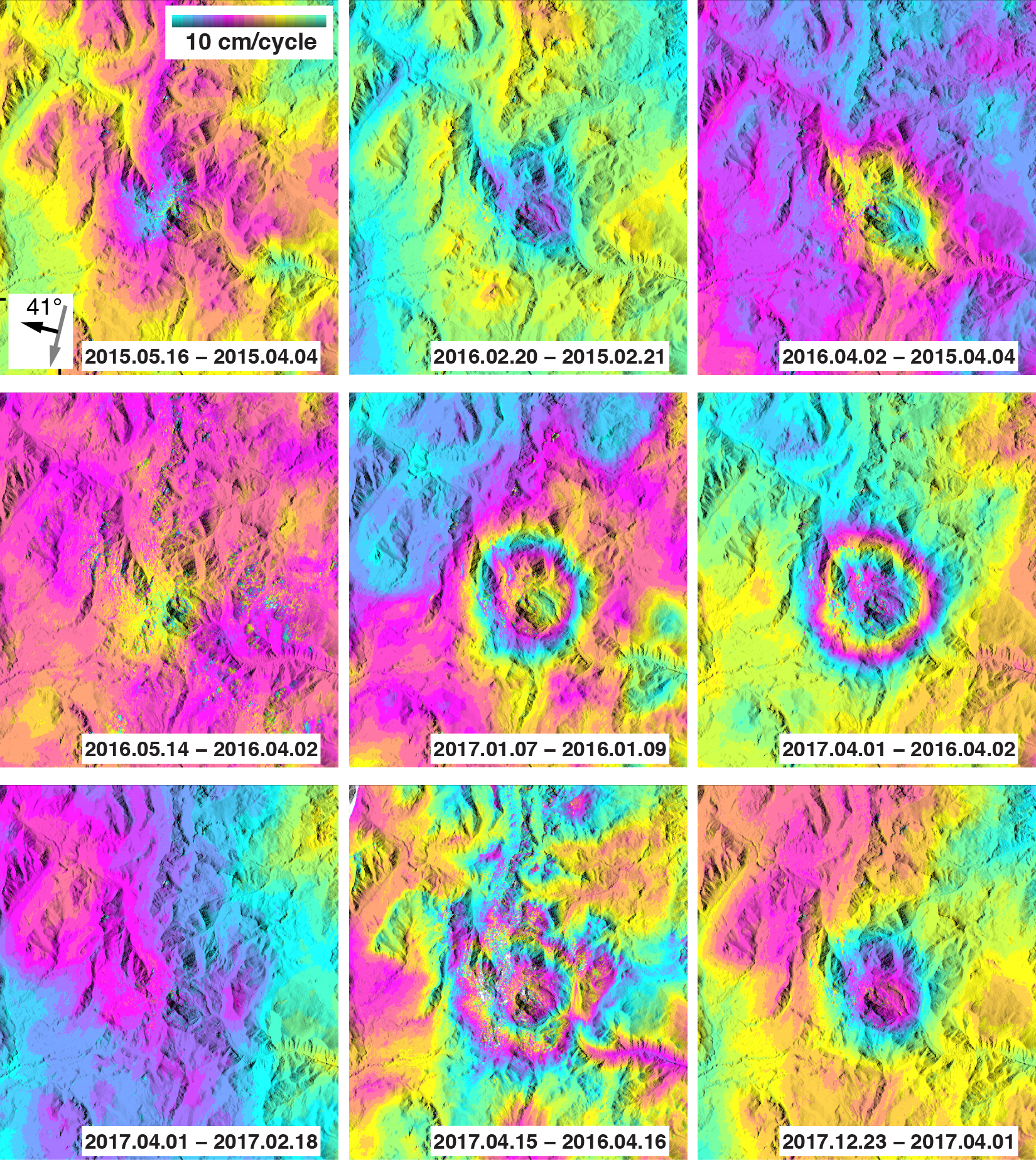


Supplementary Figure 5a. ALOS-2 SAR interferograms analyzed for descending path 129.


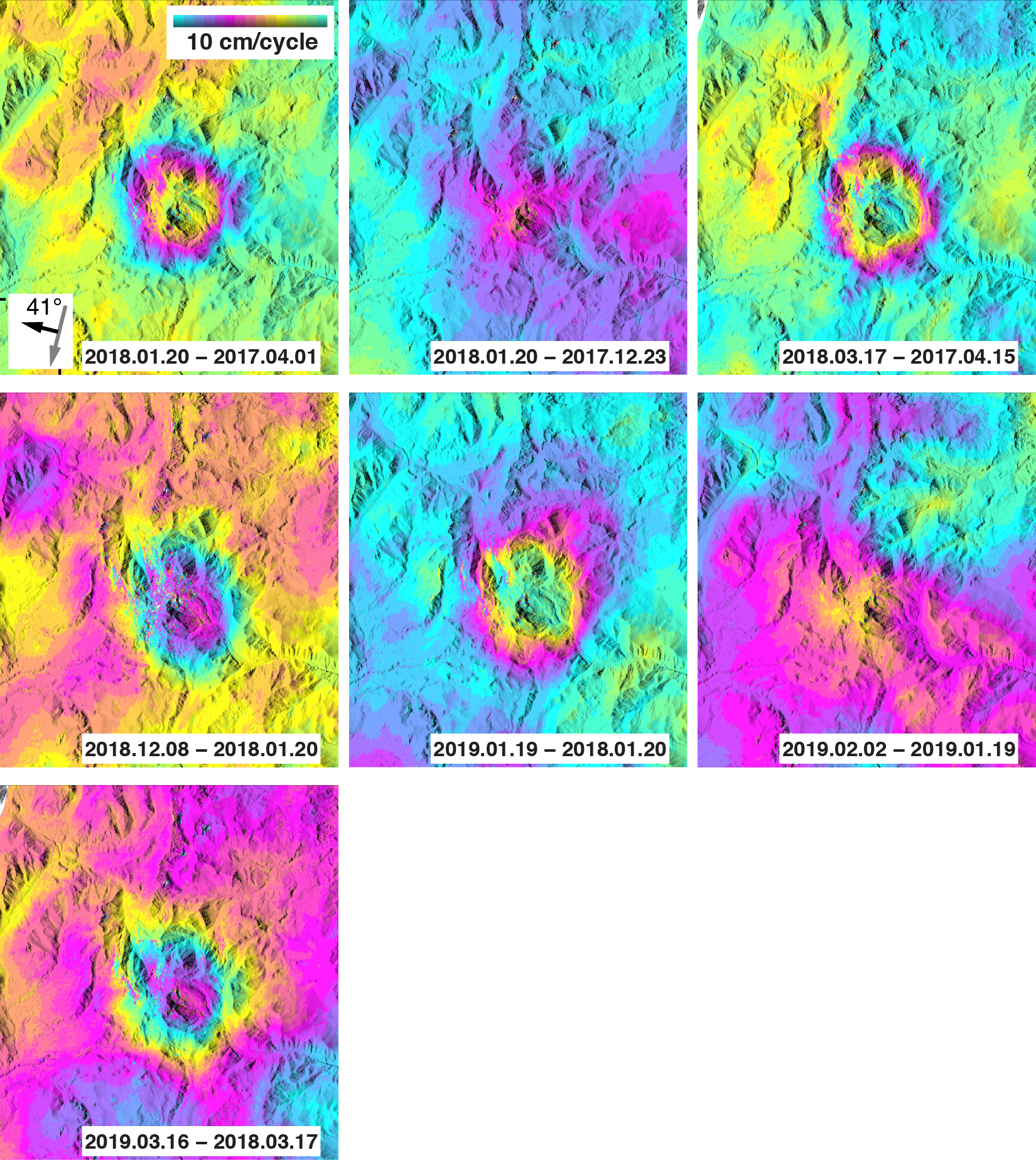


Supplementary Figure 5b. ALOS-2 SAR interferograms analyzed for descending path 129.


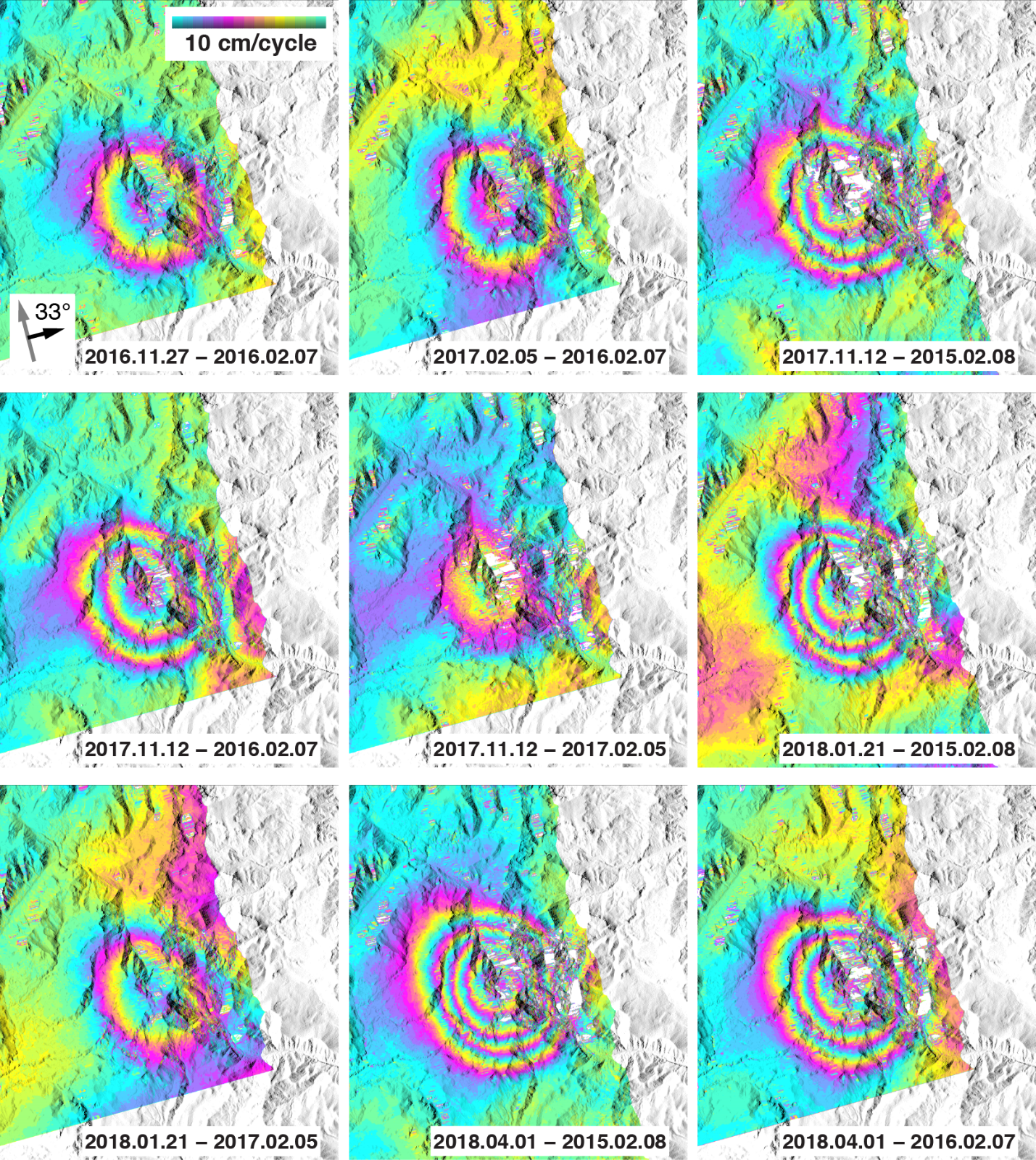


Supplementary Figure 6a. ALOS-2 SAR interferograms analyzed for ascending path 34.


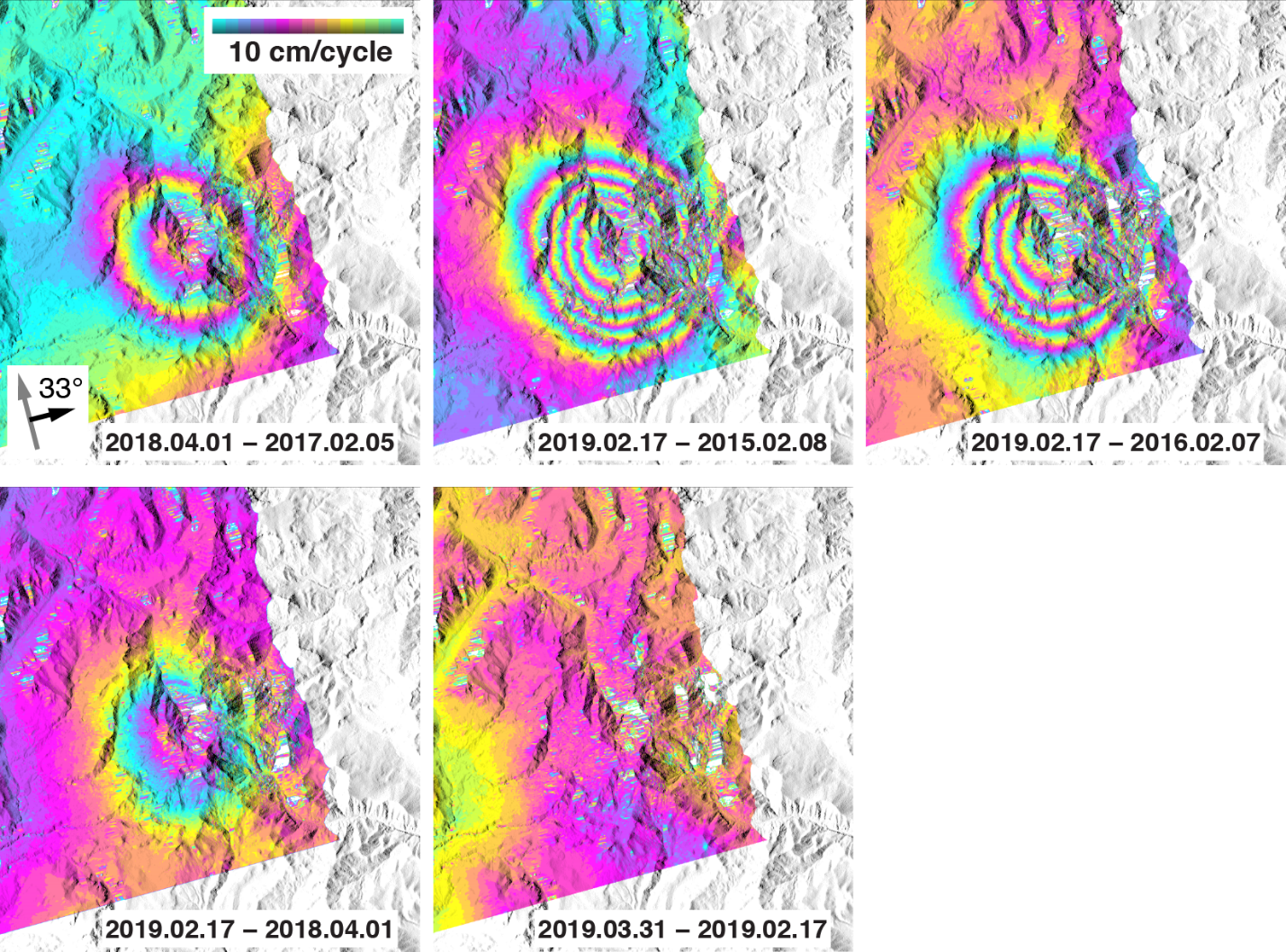


Supplementary Figure 6b. ALOS-2 SAR interferograms analyzed for ascending path 34.


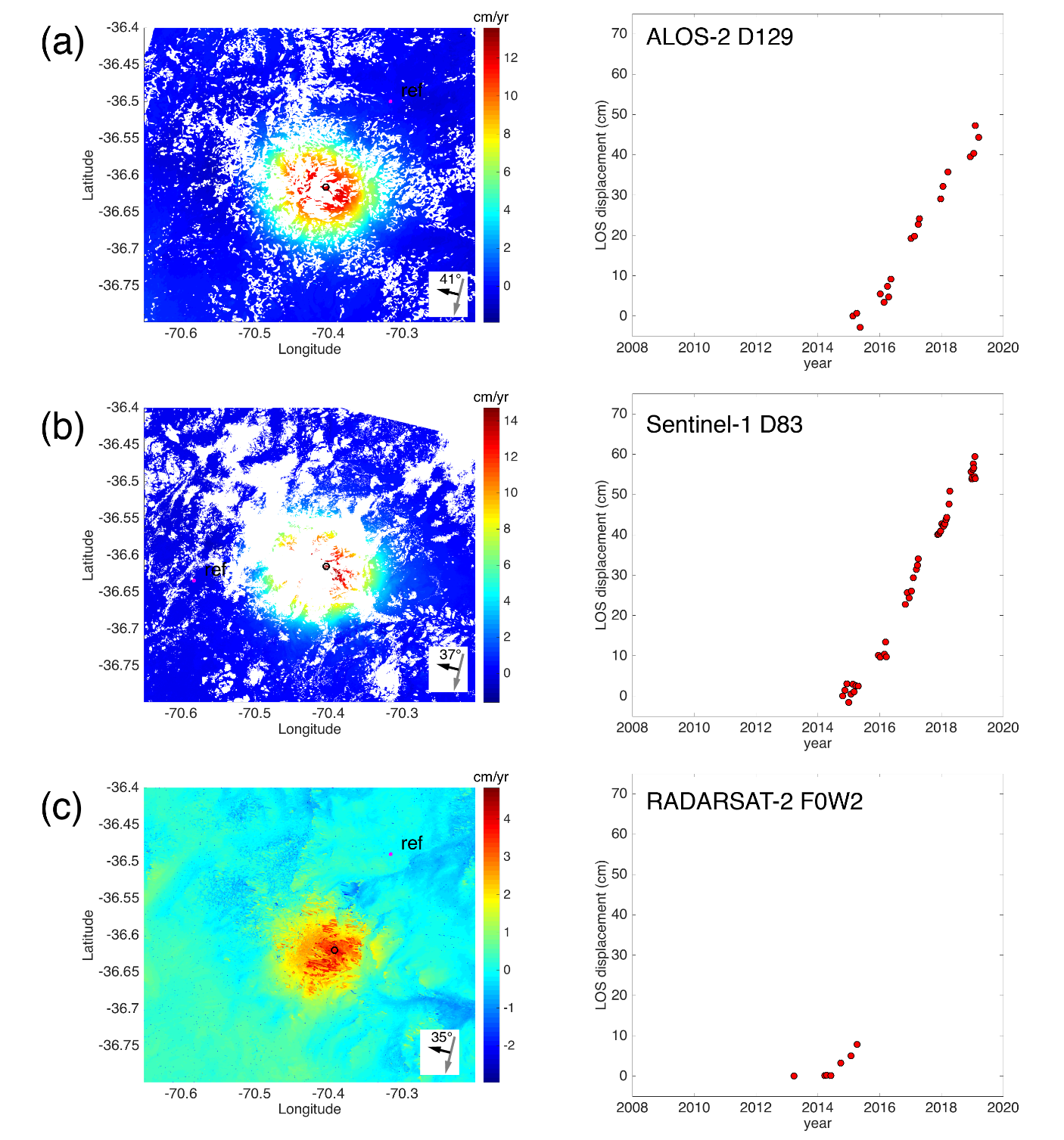


Supplementary Figure 7. InSAR time series for each satellite and track for the point given by the black circle in the linear line-of-sight (LOS) velocity map on the left, with the point time series and 2σ error bars given on the right (where large enough to be visible). The spatial reference point for each time series is shown by the magenta colored square labeled ‘ref’. The satellite heading (gray arrow) and look direction (black arrow) along with its incidence angle is shown in the lower right corner of each LOS velocity map. (a) ALOS-2 descending path 129. (b) Sentinel-1 descending track 83. (c) RADARSAT-2 descending track in F0W2 mode. Error bars are shown based on the standard deviation (σ) in LOS velocity rate for 11x11 square centered on the selected point (120 pixels) are 95% (2σ) uncertainties. Interferogram filtering (e.g. RSAT2) and very conservative retention of interferograms (e.g. throwing out interferograms that were noisy, often due to unwrapping errors in the areas of high topographic slopes) also may be responsible for very small formal errors in many cases.


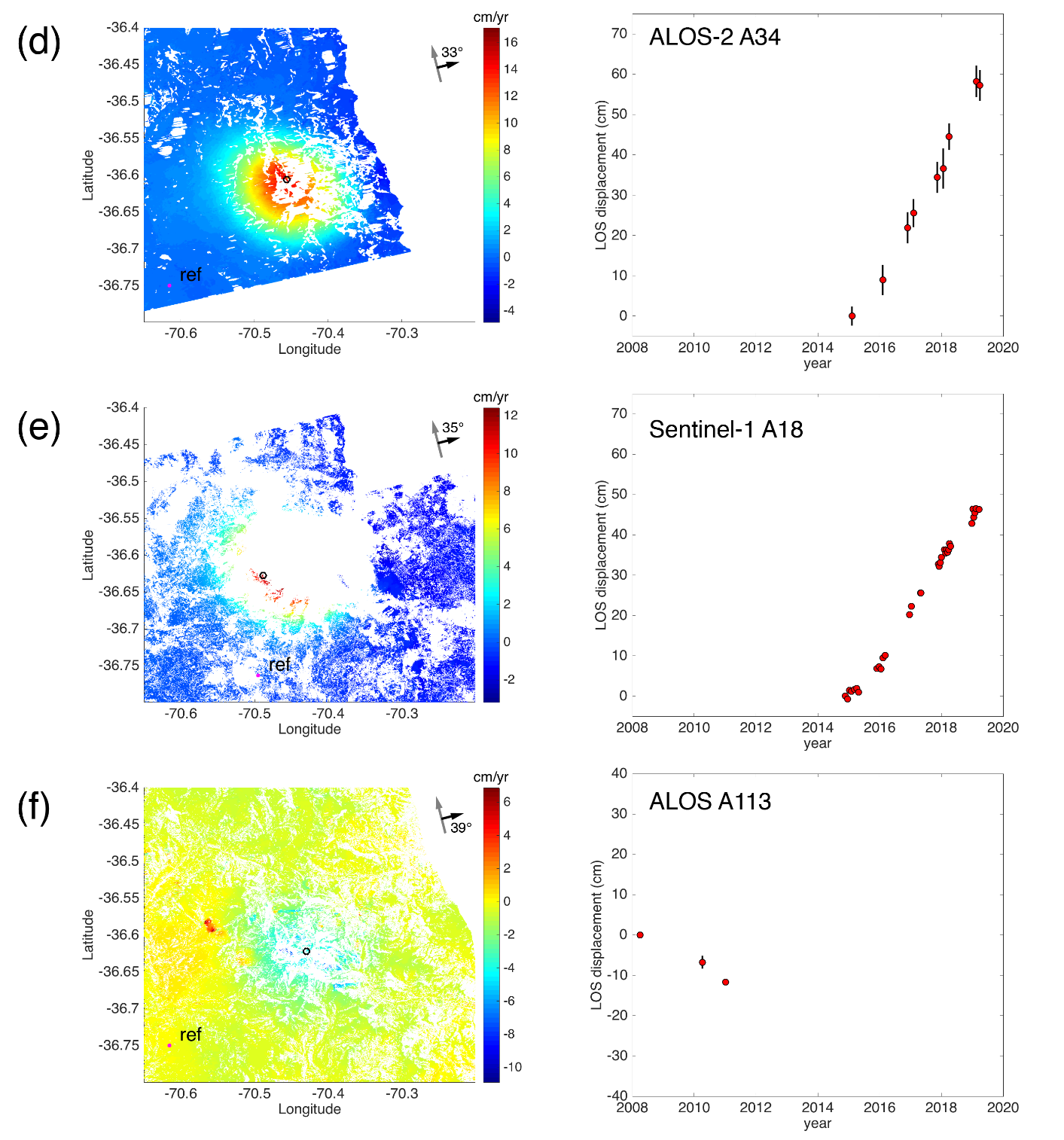


Supplementary Figure 7 (continued). InSAR time series for each satellite and track for the point given by the black circle in the linear line-of-sight (LOS) velocity map on the left, with the point time series and 2σ error bars given on the right (where large enough to be visible). The spatial reference point for each time series is shown by the magenta colored square labeled ‘ref’. The satellite heading (gray arrow) and look direction (black arrow) along with its incidence angle is shown in the lower right corner of each LOS velocity map. (d) ALOS-2 ascending path 34. (b) Sentinel-1 ascending track 18. (c) ALOS ascending track 113. Error bars are shown based on the standard deviation (σ) in LOS velocity rate for 11x11 square centered on the selected point (120 pixels) are 95% (2σ) uncertainties.


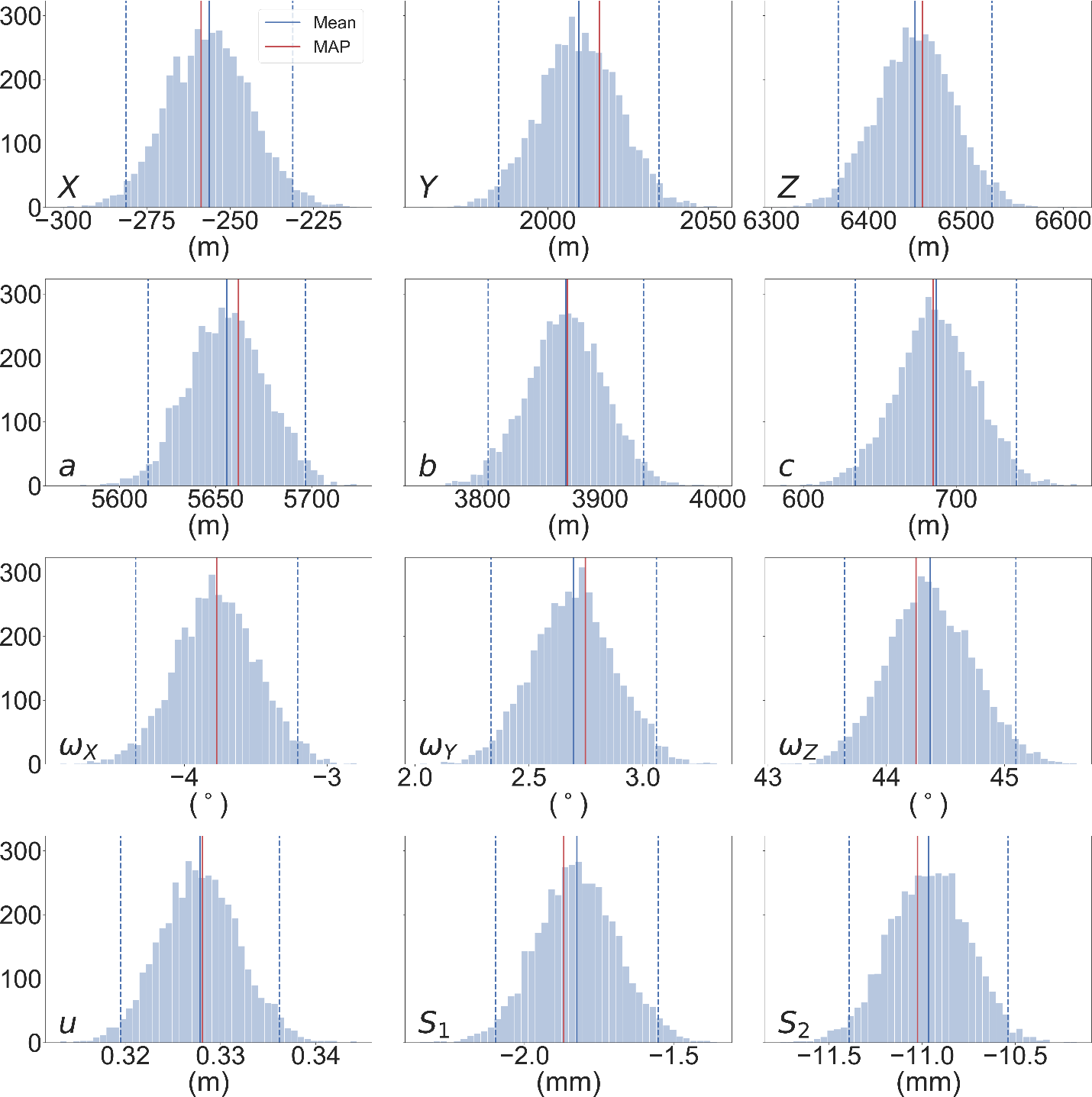


Supplementary Figure 8. Posterior, one-dimensional, probability density functions (PDFs) for the AlTar compound dislocation model^42^ (CDM) solution parameters using 4096 Markov chain Monte Carlo (MCMC) 1000 sample chains. Red vertical line shows the maximum a-posteriori (MAP) solution (‘best fit’ solution) while the blue vertical line is the mean of each PDF. Dashed vertical lines give the 95% (2σ) confidence bounds for each parameter distribution. Label in the lower left corner of each plot is the parameter name: *x*, easting location and *y* northing location, in a local cartesian coordinate system with origin at Longitude -70.43, Latitude -36.63, roughly the summit of Domuyo. Depth below the surface is given by *z*. The semi-axes lengths are given by *a, b, c*, with the axes rotation angles for each axis given by *ω_x_, ω_y_, ω_z_*. The uniform opening of each dislocation, *u*. Constant velocity offsets are given by *S_1_* and *S_2_* for the ascending and descending ALOS-2 data shown in Supplementary Figure 9, respectively.


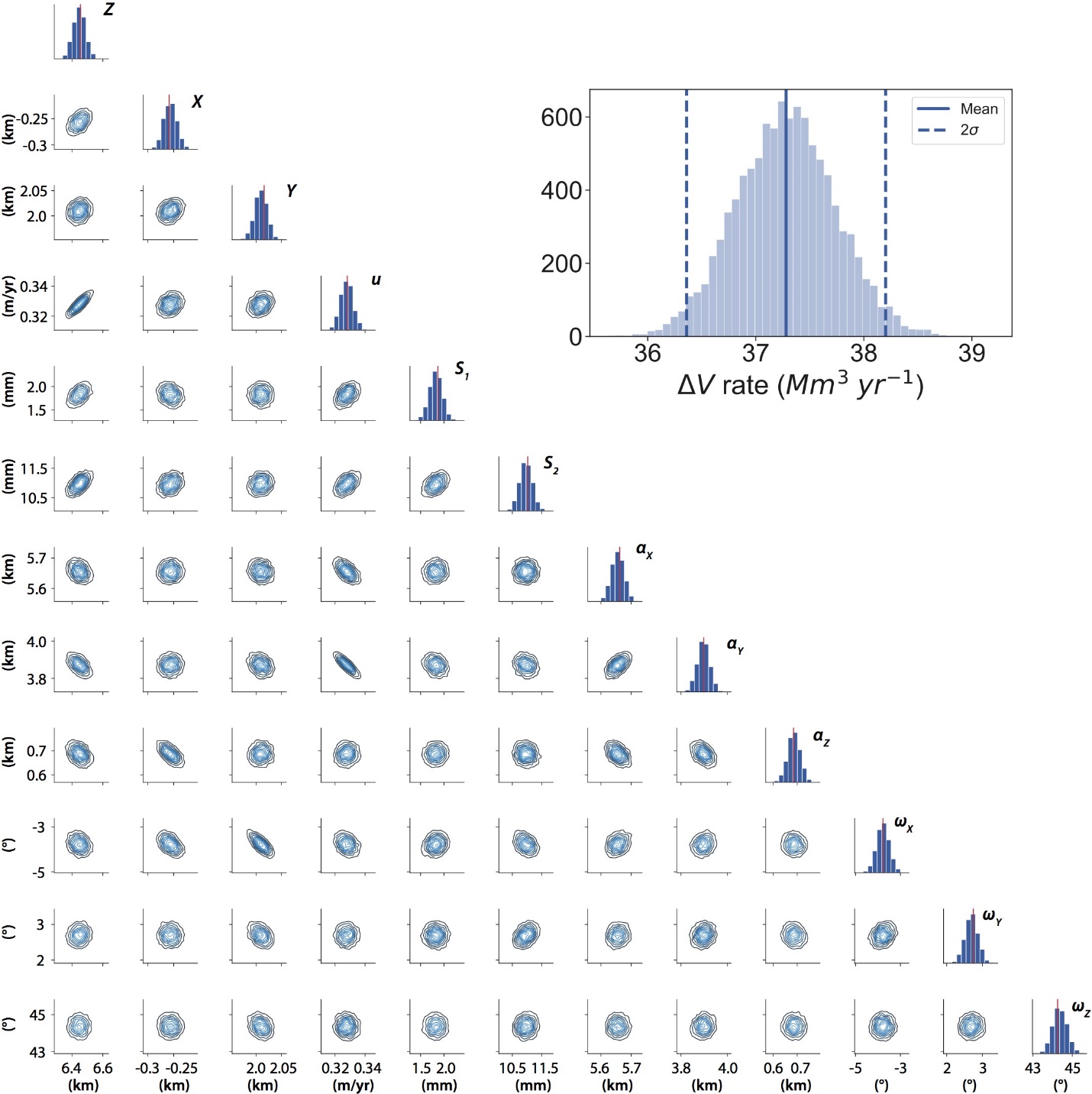


Supplementary Figure 9. Posterior probability density function (PDF) histograms for each CDM model parameter and the two-dimensional (parameter-parameter) contour plots of the sample distributions. Parameters definitions described in Supplementary Figure 8 caption. In the upper right is the volume change rate (*ΔV/Δt*) PDF generated from 10000 random draws using the PDFs of *a_x_, a_y_, a_z_, u*, where *ΔV = 4u*(*a_x_a_y_ + a_y_a_z_ + a_x_a_z_*) and *Δt is the inflation period*. The solid line is the mean and the dashed lines represent the 95% (2σ) uncertainties.


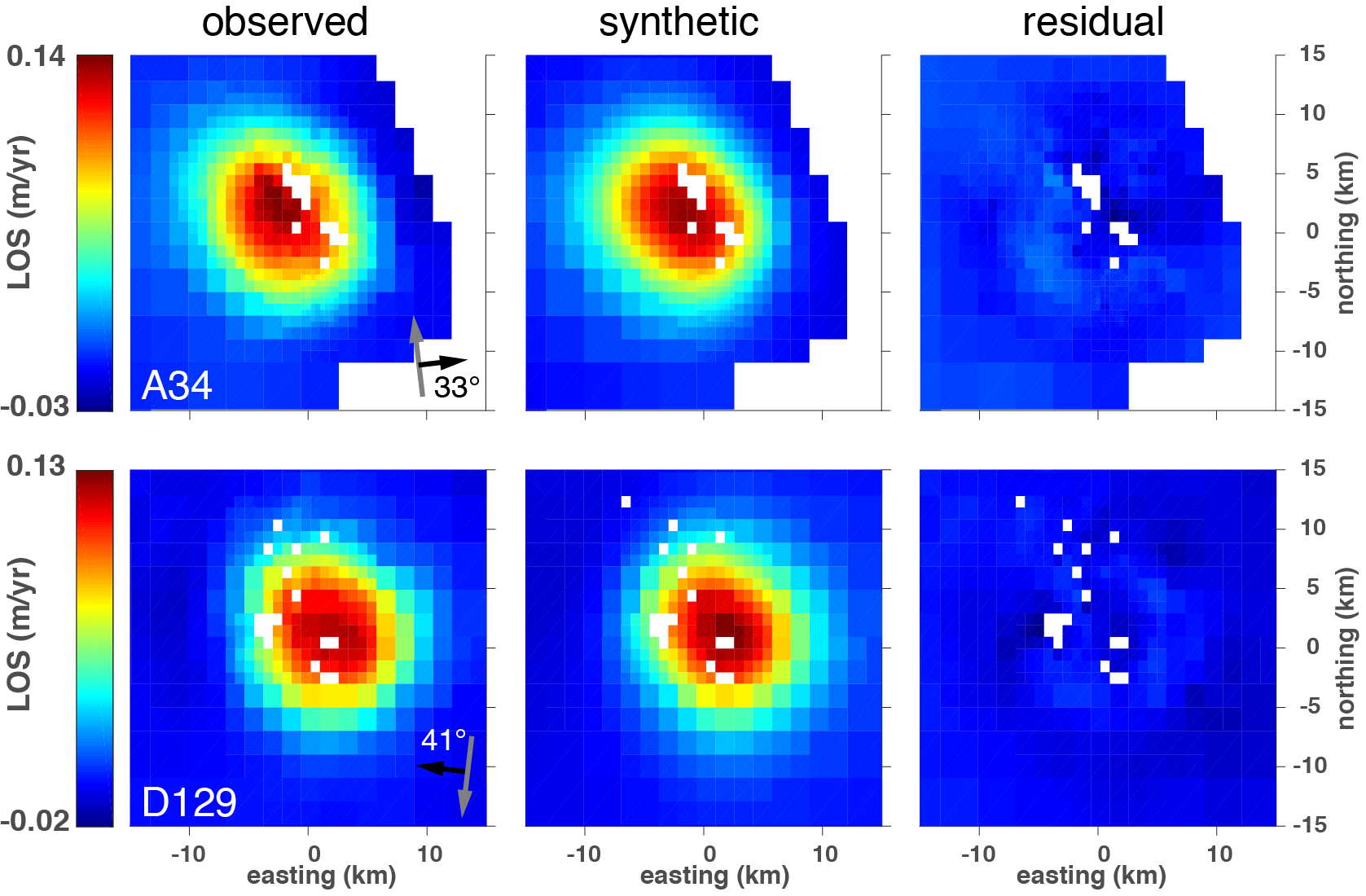


Supplementary Figure 10. Observed, synthetic, and residual for the synthetic computed from the AlTar solution shown in Figure S3 and Figure S4. The top row is for the ALOS-2 ascending path 34 down-sampled InSAR time series mean velocity and the lower row is for the ALOS-2 descending path 129 down-sampled mean velocity.


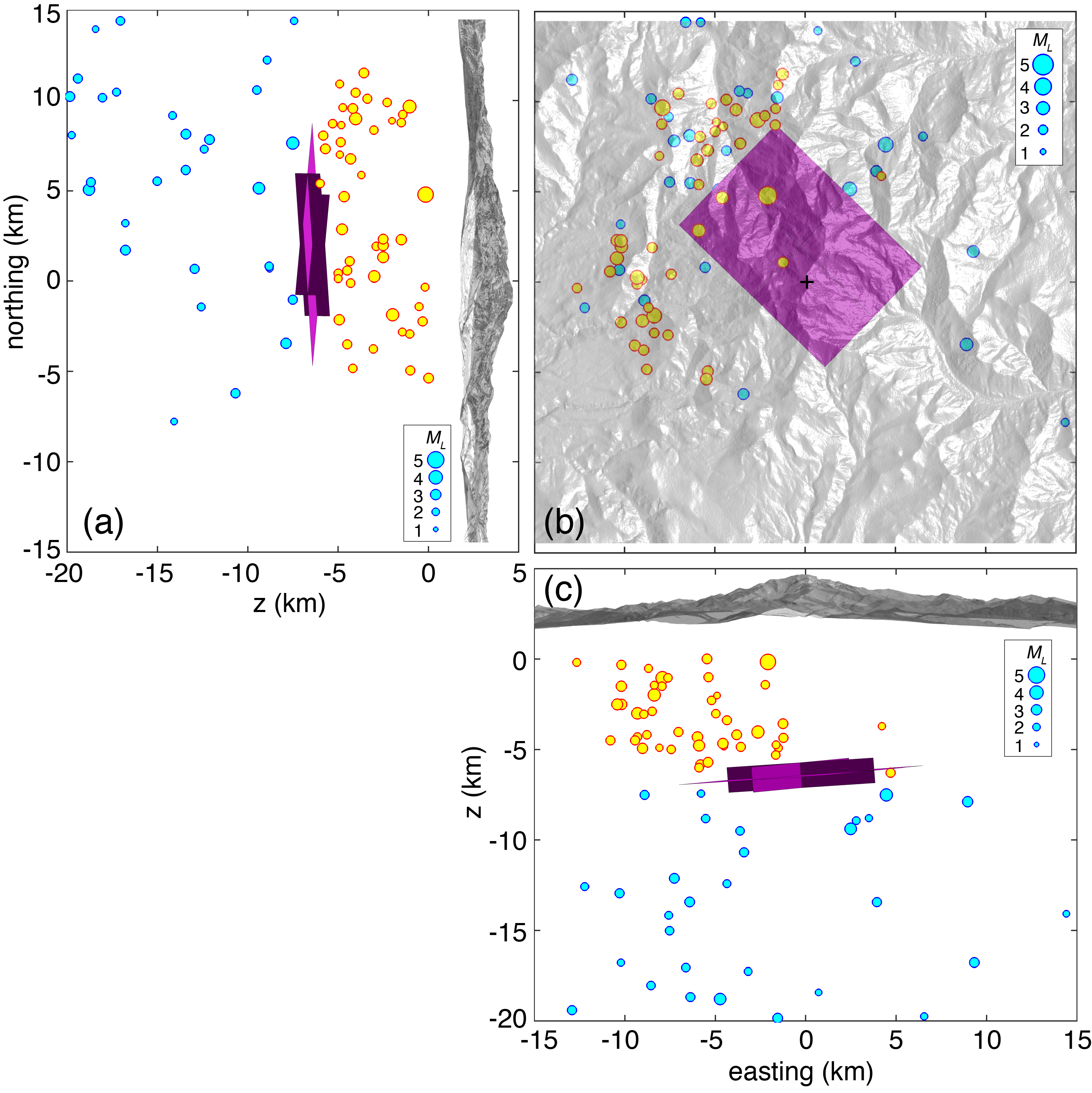


Supplementary Figure 11. AlTar source model and seismicity. (a) View from the west. (b) Map view. (c) View from the south. Seismicity above the source depth of ~6.46 km is colored yellow for identification of shallower events in (b). All depths are relative to the half-space surface.


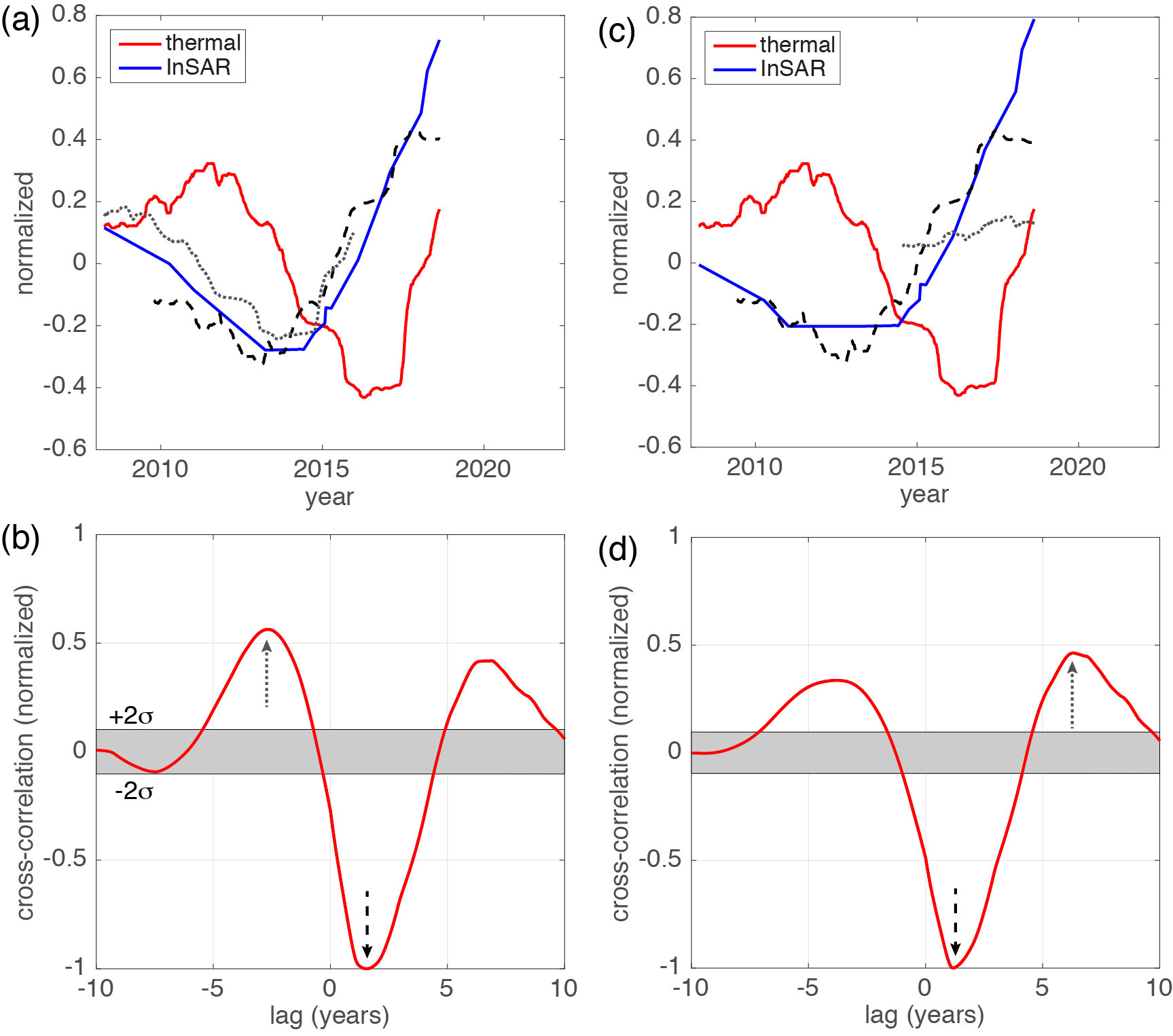


Supplementary Figure 12. Thermal and InSAR time series cross-correlation for different ALOS time series offsets. The left column plots (a, b) is for the case where the ALOS time series approaches zero deformation in early 2013, coincident with the RADARSAT-2 (RSAT2) time series initiation, which shows null deformation from 2013 - mid-2014. The right column (c, d) is for the case where the ALOS time series reaches zero at its end date (early 2011). In each column the top plot shows the normalized time series and the bottom plot shows the normalized cross-correlation. In each case the data means are removed before computing the cross-correlation. (a) Current state, InSAR time series that we have through early 2019 has been truncated at the last thermal time series date (2018.6). (b) Cross-correlation plot where the gray band gives the ±2σ (two standard deviation) uncertainty and the gray dotted and black dashed arrows indicate the shifted thermal time series in the top plot corresponding to each relative shift and scale as the two largest absolute magnitude cross-correlation peaks. (c) Same as in (a) for ALOS reaching zero deformation in early 2011. (d) Cross-correlation plot for series shown in (c). In both (a) and (c) the dashed curve shows the thermal time series plotted with the lag and scale factor corresponding to the largest negative correlation and the gray dotted line is for the thermal time series plotted with the lag and scale of the largest positive correlation peak.
